# Making water-soluble integral membrane proteins *in vivo* using an amphipathic protein fusion strategy

**DOI:** 10.1038/ncomms7826

**Published:** 2015-04-08

**Authors:** Dario Mizrachi, Yujie Chen, Jiayan Liu, Hwei-Ming Peng, Ailong Ke, Lois Pollack, Raymond J. Turner, Richard J. Auchus, Matthew P. DeLisa

**Affiliations:** 1 School of Chemical and Biomolecular Engineering, Cornell University, Ithaca, New York 14853, USA; 2 School of Applied and Engineering Physics, Cornell University, Ithaca, New York 14853, USA; 3 Department of Internal Medicine, University of Michigan, Ann Arbor, Michigan 48109, USA; 4 Department of Molecular Biology and Genetics, Cornell University, Ithaca, New York 14850, USA; 5 Department of Biological Sciences, University of Calgary, Calgary, Alberta, Canada T2N 1N4

## Abstract

Integral membrane proteins (IMPs) play crucial roles in all cells and represent attractive pharmacological targets. However, functional and structural studies of IMPs are hindered by their hydrophobic nature and the fact that they are generally unstable following extraction from their native membrane environment using detergents. Here we devise a general strategy for *in vivo* solubilization of IMPs in structurally relevant conformations without the need for detergents or mutations to the IMP itself, as an alternative to extraction and *in vitro* solubilization. This technique, called SIMPLEx (solubilization of IMPs with high levels of expression), allows the direct expression of soluble products in living cells by simply fusing an IMP target with truncated apolipoprotein A-I, which serves as an amphipathic proteic ‘shield' that sequesters the IMP from water and promotes its solubilization.

## Abstract (web summary)

The study of integral membrane proteins (IMPs) is hampered by yields and the difficulty in retaining activity once they have been solubilized. Here Mizrachi *et al.* develop a strategy for *in vivo* expression and solubilization of IMPs in functionally relevant states by fusing them to truncated apolipoprotein A-I.

## Graphical abstract

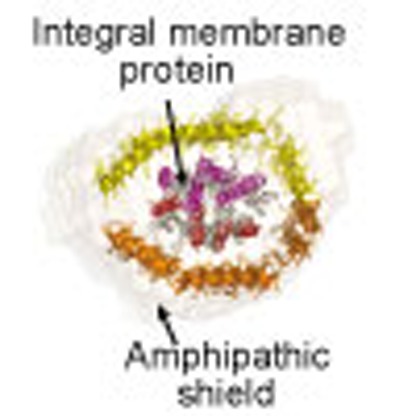

Integral membrane proteins (IMPs), which account for nearly one-third of all open reading frames in sequenced genomes^1^, play vital roles in all cells including intra- and intercellular communication and molecular transport. Given their centrality in diverse cellular functions, IMPs have enormous significance in disease^2^,^3^,^4^ and drug development^5^,^6^,^7^. However, our understanding of this important class of proteins is hampered in part by a lack of generally applicable methods for overexpression and purification, two critical steps that typically precede functional and structural analysis.

Most IMPs are naturally of low abundance and must be overproduced using recombinant systems^8^,^9^. However, the yields of chemically and conformationally homogenous, active protein following overexpression in bacteria, yeast, insect cells or cell-free systems are often still too low to support functional and/or structural characterization, and can be further confounded by aggregation and precipitation issues. This limitation can sometimes be overcome using protein engineering whereby fusion partners are used to increase expression and promote membrane integration^10^. Alternatively, mutations can be introduced to the IMP itself that enhance its stability^11^ or even render it water soluble^12^. However, these approaches are largely trial and error, and the identification of suitable fusion partners or stabilizing mutations is neither trivial nor generalizable. Even when appropriate yields can be obtained, the hydrophobic nature of IMPs requires their solubilization in an active form, which is achieved mainly through the use of detergents that strip the protein from its native lipid environment and provide a lipophilic niche inside a detergent micelle. Because IMPs interact uniquely with each detergent, identifying the best detergents often involves lengthy and costly trials. A number of detergent-like amphiphiles have been developed that stabilize IMPs in solution including protein-based nanodiscs^13^, peptide-based detergents and nanostructures^14^,^15^,^16^,^17^, amphiphilic polymers^18^ and others^19^,^20^. While these have helped to increase our knowledge of IMPs, each type of amphiphile has its own limitations, and no universal reagent has been developed for wide use with structurally diverse IMPs.

Amphipathic proteins display both hydrophilic and hydrophobic surfaces and are often associated with lipids as membrane anchors or involved in their transport as soluble particles. One example is the major component of high-density lipoprotein named apolipoprotein A-I (ApoAI), which avidly binds phospholipid molecules and organizes them into soluble bilayer structures or discs that readily accept cholesterol. ApoAI contains a globular amino-terminal (N-terminal) domain (residues 1–43) and a lipid-binding carboxyl-terminal (C-terminal) domain (residues 44–243). Biophysical studies suggest that ApoAI exhibits remarkable structural flexibility^21^, adopting a molten globular-like state for lipid-free apoA-1 under near-physiological conditions that may allow it to adapt to the significant geometry changes of the lipids with which it interacts. In support of this flexibility, truncation variants of human ApoAI lacking its 43-residue globular N-terminal domain (hereafter ApoAI*) have the ability to form nanodiscs into which detergent-solubilized IMPs can partition^13^. On the basis of this observation, we hypothesize that ApoAI* could promote soluble expression of an IMP fusion partner by providing a molecular ‘shield' that effectively sequesters the large lipophilic surfaces of the IMP from water. To test this hypothesis, we design chimeras in which ApoAI* is genetically fused to the C terminus of an IMP target. Expression of these chimeras in the cytoplasm of *Escherichia coli* yields appreciable amounts of globular, water-soluble IMPs that are stabilized in a hydrophobic environment and retain structurally relevant conformations. The approach, which we call SIMPLEx (solubilization of IMPs with high levels of expression), provides a facile method for efficiently solubilizing structurally diverse IMPs as a prelude to functional and structural studies, all without the need for detergents or lipid reconstitutions. Using SIMPLEx, we are able to study in detail a bacterial and human IMP at both the biochemical and biophysical level.

## Results

### Amphipathic ApoAI* renders bacterial EmrE water soluble

Membrane proteins are classified structurally as β-barrel or α-helical bundles. β-barrels are typically expressed as inclusion bodies, purified and refolded for structural studies, whereas α-helical bundles are less likely to produce soluble active forms after refolding. To demonstrate the SIMPLEx concept, we chose a small (110 amino acids) polytopic α-helical IMP from *E. coli* named ethidium multidrug resistance protein E (EmrE), which is comprised of four transmembrane α-helices having 18–22 residues per helix with very short extramembrane loops. EmrE is the archetypical member of the small multidrug resistance protein family in bacteria and confers host resistance to a wide assortment of toxic quaternary cation compounds by secondary active efflux^22^.

**Figure 1:**
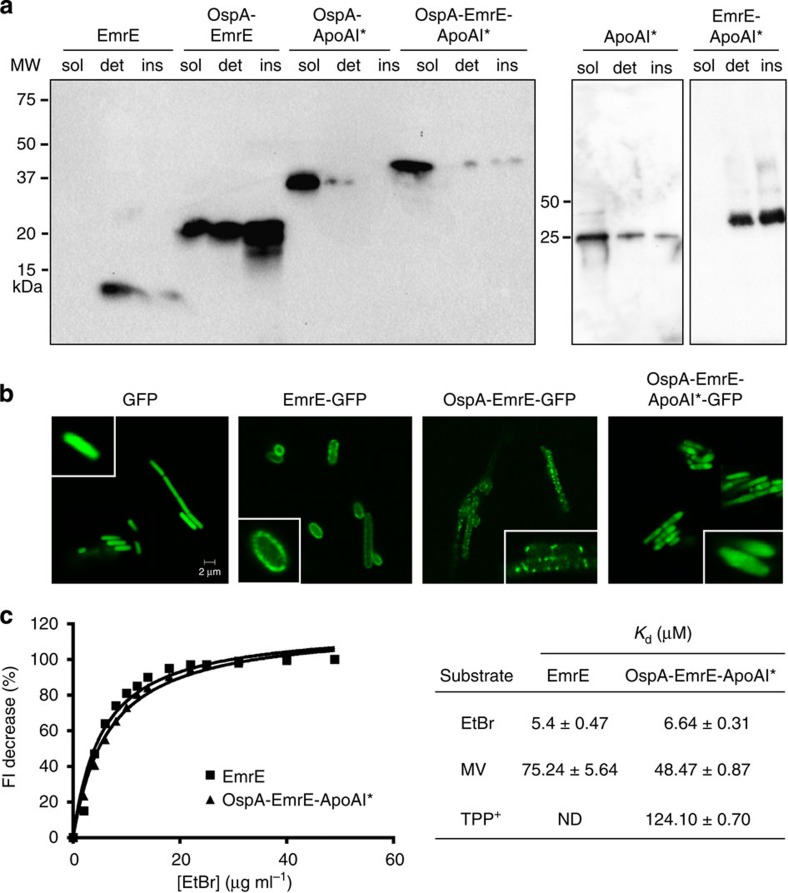
*In vivo* solubilization of EmrE using the SIMPLEx strategy. (**a**) Western blot analysis of soluble (sol), detergent solubilized (det) and insoluble (ins) fractions prepared from *E. coli* strain BL21(DE3) expressing either EmrE, OspA-EmrE, OspA-ApoAI* or OspA-EmrE-ApoAI* as indicated. Blot was probed with anti-His antibody. Molecular weight (MW) markers are shown on the left. (**b**) Fluorescence microscopy of BL21(DE3) cells expressing the same constructs in (**a**) that were each modified with a C-terminal GFP fusion for visualizing protein expression and localization. (**c**) Ligand-binding activity performed using dimeric, detergent-free OspA-EmrE-ApoAI* or organic-extracted detergent-solubilized EmrE, both of which were purified from BL21(DE3) cells. Assays were performed with EtBr as substrate. Determination of binding constants was based on fluorescence quenching. Data are expressed as the mean of biological quadruplicates and the error, defined as the s.e.m., was <5%.

To solubilize EmrE, a plasmid was created encoding a chimeric protein in which ApoAI* was fused to the C terminus of EmrE. To prevent the secretory pathway in *E. coli* from inserting EmrE directly into the inner membrane, we introduced a highly soluble ‘decoy' protein from *Borrelia burgdorferi,* namely outer surface protein A (OspA)^23^, to the N terminus of the EmrE-ApoAI* chimera. We predicted that the resulting tripartite fusion would partition to the cytoplasm due to the presence of the N-terminal OspA decoy and would give rise to solubilized EmrE due to the proteic shield afforded by ApoAI*. To test this hypothesis, we examined the cellular accumulation of OspA-EmrE-ApoAI* in *E. coli* cells transformed with pSIMPLEx-EmrE. Western blot analysis of the soluble cytoplasmic fraction recovered from these cells confirmed that the tripartite fusion was a stable, water-soluble protein with hardly any of the fusion protein partitioning to the insoluble fraction (Fig. 1a). Following cell lysis and Ni^2+^-affinity chromatography in the absence of detergents, we obtained ∼10–15 mg of OspA-EmrE-ApoAI* per litre of culture. Size-exclusion chromatography (SEC) confirmed that the majority of the soluble OspA-EmrE-ApoAI* was dimers and tetramers (Supplementary Fig. 1a), consistent with the earlier observation that the basic functional unit of EmrE is the dimer but may also include a dimer of dimers^24^. Peaks corresponding to dimers and tetramers were isolated and reapplied to the SEC (Supplementary Fig. 1b). Final yields of both species ranged between 8 and 10 mg l^−1^ of culture. It is worth noting that the solubility profile of OspA-EmrE-ApoAI* was nearly identical to that of a control fusion, OspA-ApoAI* lacking the IMP, which also accumulated exclusively in the soluble fraction (Fig. 1a). In stark contrast, EmrE expressed alone was detected in the detergent soluble and insoluble fractions only (Fig. 1a). A fusion comprised of OspA and EmrE without the ApoAI* domain accumulated in all three fractions of the lysate (soluble, detergent soluble and insoluble). However, all of the soluble OspA-EmrE was aggregated as confirmed by SEC (Supplementary Fig. 1c). The importance of the decoy was revealed by an EmrE-ApoAI* fusion lacking the OspA decoy, which accumulated in the detergent soluble and insoluble fractions in a manner similar to EmrE expressed alone (Fig. 1a). This insolubility was largely due to EmrE as the ApoAI* domain expressed on its own accumulated in all three fractions of the lysate (Fig. 1a).

In parallel, we also investigated whether the OspA domain could be replaced with a structurally different soluble decoy, namely *E. coli* maltose-binding protein lacking its native export signal peptide (ΔspMBP). Indeed, the tripartite ΔspMBP-EmrE-ApoAI* fusion accumulated exclusively in the soluble fraction, just like its OspA-EmrE-ApoAI* counterpart (Supplementary Fig. 2a). Hence, solubilization appears to be insensitive to the identity or structure of the N-terminal domain. Moreover, when the N-terminal domain was removed by proteolytic digestion, the resulting IMP-ApoAI* cleavage product remained soluble (Supplementary Fig. 2b). Together these results suggest that the N-terminal domain functions to direct folding away from the membrane while the ApoAI* domain promotes water solubility. Since one ApoAI* monomer is capable of binding 70–100 lipids in nanodiscs^25^, it is possible that the observed solubilization was the result of similar lipid incorporation into the fusion construct. However, when we measured the lipid content of ΔspMBP-EmrE-ApoAI*, only 5–10 lipids per monomer of ApoAI* were detected. Hence, we conclude that the solubilization of IMPs by ApoAI* is due to protein–protein interactions and not the incorporation of a large number of lipids.

### Visualization of solubilized EmrE in the cytoplasm

To determine the localization of the different EmrE chimeras, a green fluorescent protein (GFP) domain was added to the C terminus of each construct. Bacterial cells synthesizing GFP-tagged membrane proteins typically exhibit a fluorescent signal that is circumferential around the cell periphery, reflecting uniform distribution of the protein within the membrane. As expected, EmrE lacking the OspA and ApoAI* domains localized in the membrane as evidenced by uniform green fluorescence appearing at the periphery of cells expressing EmrE-GFP (Fig. 1b). In contrast, diffuse cytoplasmic fluorescence was observed for cells expressing the solubilized OspA-EmrE-ApoAI*-GFP chimera (Fig. 1b), consistent with the fluorescence patterns seen for soluble GFP-tagged proteins or for GFP expressed alone. Expression of the GFP-tagged OspA-EmrE, which lacked the solubilizing ApoAI* domain, resulted in bright punctate fluorescent foci around the periphery of the cell and throughout the cytoplasm (Fig. 1b). The uneven distribution of GFP-tagged OspA-EmrE in the membrane and its accumulation at discrete locations in the cell is indicative of protein aggregation and strongly suggestive of defects in protein folding and membrane insertion, in line with the SEC results for this fusion. This would also suggest that the presence of a third protein at the C terminus of the fusion ensemble guarantees neither the solubility nor the proper folding of the IMP. Importantly, western blot analysis confirmed that the fluorescence observed in each of these cases was due to intact GFP fusions and not proteolytically released GFP domains (Supplementary Fig. 3a).

### Solubilized EmrE passes folding quality-control checkpoint

As a final confirmation of intracellular solubility, we subjected the OspA-EmrE-ApoAI* chimera and related constructs to the folding quality-control (QC) mechanism inherent to the *E. coli* twin-arginine translocation (Tat) pathway^26^. Previous studies established that Tat QC interrogates the foldedness of its substrate proteins, allowing export of only those that are properly folded, soluble, and non-aggregated^26^,^27^,^28^. To determine whether any of the EmrE constructs could pass this discriminatory filter, each was modified at its N terminus with the archetypal Tat export signal from *E. coli* trimethylamine *N*-oxide reductase (spTorA). This 39-residue signal peptide bears a canonical twin-arginine motif (S/T-R-R-X-F-L-K) and has been extensively used to target structurally diverse recombinant proteins for proofreading by the Tat translocase^26^,^27^. Both spTorA-OspA-EmrE-ApoAI* and spTorA-OspA-ApoAI* were capable of passing the QC filter and localizing in the periplasm (Supplementary Fig. 3b), as expected for soluble, non-aggregated proteins. On the other hand, neither spTorA-EmrE nor spTorA-OspA-EmrE was exported (Supplementary Fig. 3b), in agreement with their accumulation in the insoluble fraction. In the latter case, some of the spTorA-OspA-EmrE was detected in the soluble fraction, but the lack of any Tat export further suggests that the soluble OspA-EmrE is predominantly higher molecular weight aggregates that are blocked for Tat export. In all cases, detection of the cytoplasmic chaperone GroEL confirmed the integrity of fractionation (Supplementary Fig. 3b). It should be pointed out that the overall expression level of spTorA-OspA-EmrE-ApoAI* detected in these experiments was visibly lower. While this could arise from poor translation and/or poor mRNA stability of the artificial fusion sequence, we suspect that the lower expression is related to poor translocation efficiency. Even though spTorA-OspA-EmrE-ApoAI* appears to be a well-folded, soluble substrate, its export efficiency could be impeded due to its large cross-sectional area, which has been proposed as a limiting variable in the export of folded proteins by the Tat system. It is well documented that inefficiently translocated substrates are efficiently degraded as part of a poorly characterized ‘housecleaning' mechanism associated with that Tat system^26^,^27^, which could thus account for the lower total expression of spTorA-OspA-EmrE-ApoAI*.

### Solubilized EmrE retains ligand-binding activity

We next determined whether EmrE that had been solubilized by fusion to ApoAI* was able to bind to known ligands. Native EmrE transports and thus binds several substrates including ethidium bromide (EtBr), methyl viologen (MV) and tetraphenylphosphonium (TPP^+^)^22^. There are a total of 14 aromatic amino acids in EmrE (four tryptophans, five tyrosines and five phenylalanines), some of which participate in ligand binding and protein stability and permit determination of binding constants based on fluorescence quenching^22^. The ligand-binding activity of dimeric, detergent-free OspA-EmrE-ApoAI* was measured and compared with detergent-solubilized EmrE. Remarkably, the binding affinity of water-soluble OspA-EmrE-ApoAI* for EtBr was very similar to that measured for detergent-solubilized EmrE, while the affinities for MV and TPP^+^ were each higher for ApoAI*-solubilized EmrE compared with its detergent-solubilized counterpart (Fig. 1c and Supplementary Fig. 4). Importantly, ApoAI* alone showed no measurable binding of any of the ligands (data not shown). In light of these results, it should be pointed out that the environment in which ligand-binding activity of EmrE is measured plays a crucial role, with binding affinity varying as a function of the membrane mimetic employed^29^. For instance, the *K*_d_ values calculated for *in vitro* solubilized EmrE and MV were 38.2, 5.4 and 46.2 μM when measured in small unilamellar vesicles, SDS or dodecylmaltoside, respectively. For *in vivo*-solubilized EmrE, we obtained a value of 48 μM, which compares favourably with small unilamellar vesicles. On the other hand, *K*_d_ values for EtBr were similar among the three membrane mimetics and for *in vivo*-solubilized EmrE (∼5.5 μM). Thus, the fact that water-soluble OspA-EmrE-ApoAI* exhibits ligand-binding activity with kinetic constants that is on par with native EmrE suggests that *in vivo-*solubilized IMPs can be folded into a functional form.

### Solubilization of human cytochrome *b*_5_ by ApoAI*

**Figure 2:**
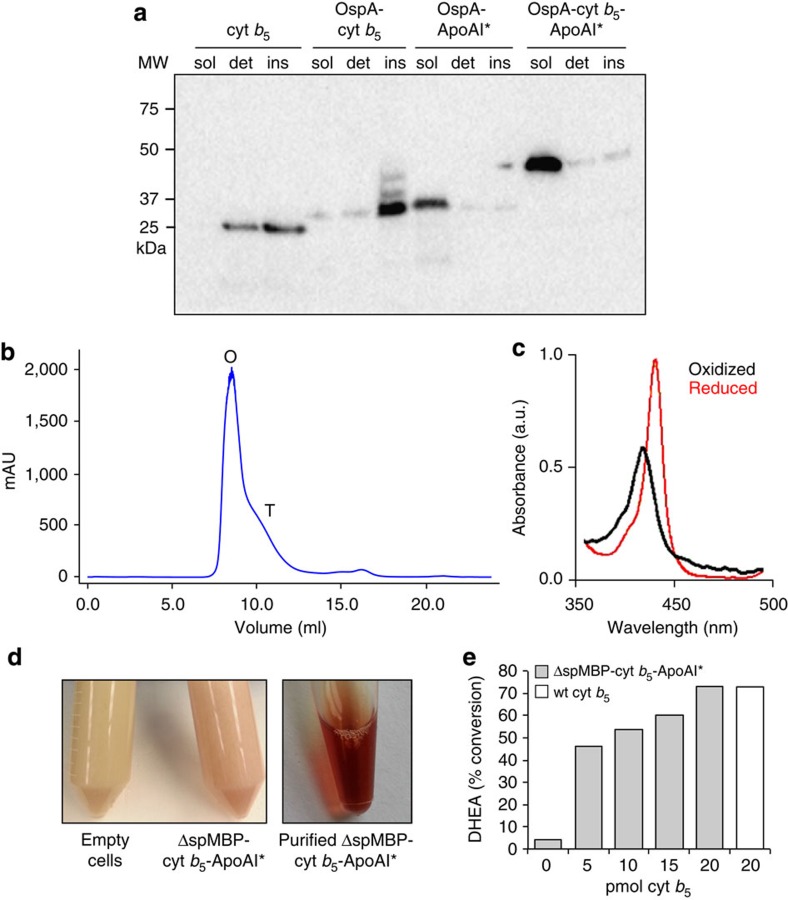
*In vivo* solubilization of cyt *b*_5_ using the SIMPLEx strategy. (**a**) Western blot analysis of soluble (sol), detergent solubilized (det) and insoluble (ins) fractions prepared from *E. coli* strain BL21(DE3) expressing either cyt *b*_5_, OspA-cyt *b*_5_, OspA-ApoAI* or OspA-cyt *b*_5_-ApoAI* as indicated. Blot was probed with anti-His antibody. Molecular weight (MW) markers are shown on the left. (**b**) SEC profiles of Ni^2+^-purified ΔspMBP-cyt *b*_5_-ApoAI*. Protein elutes as an octamer (O) or tetramer (T). (**c**) Oxidized and reduced spectra of ΔspMBP-cyt *b*_5_-ApoAI* with characteristic peaks at 424 and 409 nm, respectively. (**d**) Coordination of haeme cofactor by cyt *b*_5_. Cells carrying empty plasmid control or expressing ΔspMBP-cyt *b*_5_-ApoAI* were visually inspected for characteristic red haeme colouring. Purified ΔspMBP-cyt *b*_5_-ApoAI* is also shown. (**e**) Augmentation of CYP17A1 lyase activity by cyt *b*_5_. CYP17A1 and its substrate 17-hydroxy pregnenolone (17-P5) were incubated with different concentrations (5–25 pmol) of wild-type cyt *b*_5_ (detergent solubilized) or ΔspMBP-cyt b5-ApoAI*, or in the absence of cyt *b*_5_. The percentage of dehydroepiandrosterone (DHEA) product formed was monitored by high-performance liquid chromatography.

**Figure 3:**
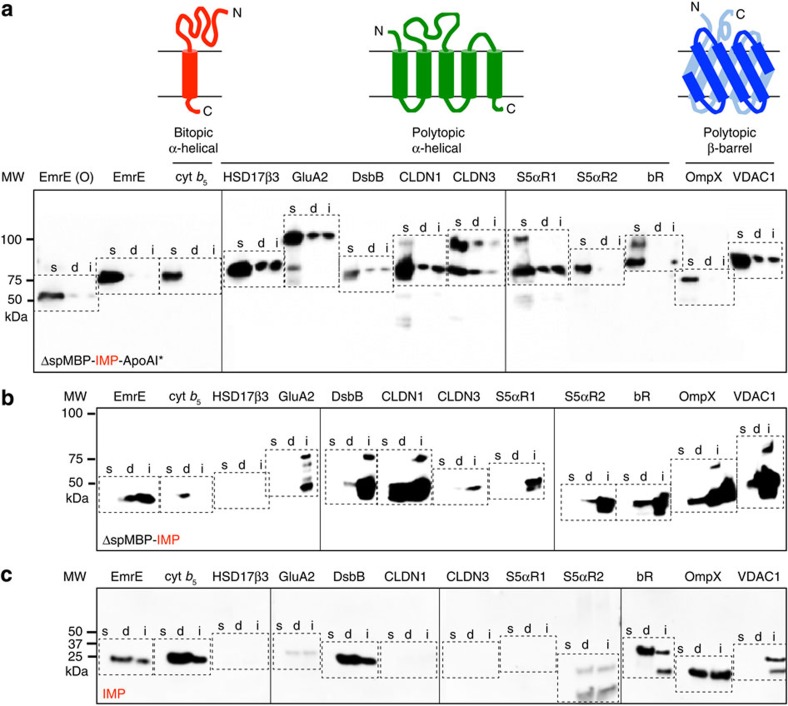
*In vivo* solubilization of structurally diverse IMP targets. Western blot analysis of soluble (s), detergent solubilized (d) and insoluble (i) fractions prepared from *E. coli* strain BL21(DE3) expressing: (**a**) ΔspMBP-IMP-ApoAI*; (**b**) ΔspMBP-IMP; or (**c**) IMP alone. The IMP targets included (from left to right): (lanes 1–3) EmrE with OspA decoy (O); (lanes 4–6) EmrE with ΔspMBP decoy; (lanes 7–9) *H. sapiens* cyt *b*_5_; (lanes 10–12) *H. sapiens* HSD17B3; (lanes 13–15) *H. sapiens* GluA2; (lanes 16–18) *E. coli* DsbB; (lanes 19–21) *H. sapiens* CLDN1; (lanes 22–24) *H. sapiens* CLDN3; (lanes 25–27) *H. sapiens* S5aR1; (lanes 28–30) *H. sapiens* S5αR2; (lanes 31–33) *Halobacterium sp.* NRC-1 bR; (lanes 34–36) *E. coli* OmpX; and (lanes 37–39) *R. norvegicus* VDAC1. Dashed-line boxes denote the soluble, detergent solubilized and insoluble fractions prepared for each IMP target. Blots were probed with anti-His antibody. Molecular weight (MW) markers are shown on the left.

Encouraged by the ability of ApoAI* to solubilize the polytopic bacterial EmrE, we tested whether a structurally unrelated IMP, namely human cytochrome *b*_5_ (cyt *b*_5_), could be similarly solubilized. Cyt *b*_5_ is a 134-residue bitopic membrane protein consisting of six α-helices and five β-strands folded into three distinct domains: (i) an N-terminal haeme-containing soluble domain; (ii) a C-terminal membrane anchor; and (iii) a linker or hinge region that connects the two domains. Solubility trials of OspA-cyt *b*_5_-ApoAI* resulted in an identical pattern of solubility as seen for OspA-EmrE-ApoAI* (Fig. 2a). Like EmrE, cyt *b*_5_ was similarly solubilized when the OspA decoy was replaced by ΔspMBP (Fig. 3a, lanes 7–9). The addition of ΔspMBP and ApoAI* did not affect homo-oligomer formation as solubilized ΔspMBP-cyt *b*_5_-ApoAI* was predominantly octameric (Fig. 2b), consistent with the oligomerization state of the detergent-solubilized enzyme^30^. Solubilization also did not appear to disrupt haeme cofactor acquisition as evidenced by the visibly red color of cells expressing ΔspMBP-cyt *b*_5_-ApoAI* and of purified ΔspMBP-cyt *b*_5_-ApoAI* (Fig. 2d), as well as by the prototypical reduced and oxidized spectra obtained for purified ΔspMBP-cyt *b*_5_-ApoAI* at 424 and 409 nm, respectively (Fig. 2c). Yields of *in vivo*-solubilized cyt *b*_5_ were 5–8 mg l^−1^ of culture.

Since cofactor incorporation is obligatory for function of this IMP, we next tested whether solubilized cyt *b*_5_ was functional. Native cyt *b*_5_ stimulates the 17,20-lyase activity of cytochrome P450c17 (17α-hydroxylase/17,20-lyase; CYP17A1). In particular, a molar equivalent of cyt *b*_5_ increases the rate of the 17,20-lyase reaction 10-fold, via an allosteric mechanism that does not require electron transfer^31^. The ability of ΔspMBP-cyt *b*_5_-ApoAI* to stimulate lyase activity of CYP17A1 was assayed *in vitro* and compared with wild-type cyt *b*_5_ that had been detergent solubilized. Importantly, ΔspMBP-cyt *b*_5_-ApoAI* stimulated lyase activity in a dose-dependent manner (Fig. 2e and Supplementary Fig. 5). At these same concentrations and conditions, the stimulatory activity measured for the detergent-solubilized cyt *b*_5_ was plateaued (Supplementary Fig. 5); however, at slightly lower concentrations, detergent-solubilized cyt *b*_5_ also showed dose-dependent stimulation (data not shown) consistent with previous findings^32^. Hence, the detergent-solubilized cyt *b*_5_ was a slightly better stimulator of lyase activity than the *in vivo*-solubilized version. Nonetheless, both enzymes were able to promote maximal stimulatory activity under the conditions tested here. Given that the C-terminal transmembrane helix of cyt *b*_5_ is required to stimulate the 17,20-lyase activity of human CYP17A1 (ref. 33), we conclude that the ApoAI* shield must be sufficiently flexible to allow the protein–protein interactions that are necessary to promote proper function.

### Solubilization of structurally diverse IMPs using SIMPLEx

We next sought to extend the SIMPLEx technique to a panel of 10 additional IMP targets including: polytopic α-helical IMPs comprised of three (*Homo sapiens* hydroxy steroid dehydrogenase, HSD17β-3), four (*E. coli* DsbB; *H. sapiens* glutamate receptor A2, GluA2; *H. sapiens* Claudin1, CLDN1; and *H. sapiens* Claudin3, CLDN3), five (*H. sapiens sapiens* steroid 5α-reductase types 1 and 2, S5αR1 and S5αR2) or seven (*Halobacterium sp.* NRC-1 bacteriorhodopsin, bR) transmembrane helices; and polytopic β-barrel IMPs (*E. coli* OmpX and *Rattus norvegicus* voltage-dependent anion channel 1, VDAC1). Using the ΔspMBP-IMP-ApoAI* format, all ten of these IMP targets were produced at significant levels in the soluble fraction in the absence of detergents (Fig. 3a and Supplementary Fig. 6). While some of the IMP targets were also detected in the insoluble fraction, the amount of IMP partitioned in the soluble fraction was significantly higher in every case. As expected, none of the IMPs was detected in the soluble fraction when ApoAI* or both ΔspMBP and ApoAI* were omitted from the fusion (Fig. 3b,c, respectively). Instead, these constructs typically partitioned to the detergent soluble and/or insoluble fractions. Moreover, for a subset of these control constructs, namely those involving HSD17β-3, GluA2, CLDN3, S5αR1 and S5αR3, little to no expression was observed in the detergent soluble fractions (Fig. 3b,c). Only when these IMPs were expressed in the SIMPLEx format were they rendered soluble, suggesting that SIMPLEx is a more general strategy for creating water-soluble versions of structurally diverse IMPs.

### Structural characterization of solubilized EmrE

An important question is whether IMPs solubilized by the SIMPLEx strategy are amenable to structural characterization. To answer this question, we focused our attention on the EmrE protein. First, negative staining electron microscopy was used to observe dimeric OspA-EmrE-ApoAI* in solution. The analysis revealed a homogeneous population of monodisperse proteins (Supplementary Fig. 7). A small number of larger-sized particles, deviating from the average size of ∼5–15 nm, were observed that may represent different orientations of the soluble particles or traces of tetramers that were incompletely removed during purification.

Second, dynamic light scattering was used to obtain information about the size and behaviour of our fusion protein in solution. Specifically, we evaluated how the solution behaviour of dimeric OspA-EmrE-ApoAI* changed in the absence and presence of one of its ligand, EtBr. Compared with the fusion protein in buffer alone, exposure to increasing amounts of EtBr resulted in a clear shift to higher molecular masses within a short period of time (∼15 min, Supplementary Fig. 8a–c). When the same fusion protein was exposed to increasing amounts of CHAPS detergent that exceeded its critical micelle concentration, the protein size did not change over time (>1 h; Supplementary Fig. 8d–f). Taken together, these data reveal a possible conformational transition from a ligand-free dimer unit to a ligand-bound higher-degree oligomer formed by two dimers (that is, dimer of dimers)^24^. Moreover, the ability of ApoAI* to not only solubilize EmrE but also to accommodate its native protein–protein interactions (that is, dimer formation) all within the fusion context suggests a remarkable plasticity for this amphipathic domain.

Third, biological small-angle X-ray scattering (SAXS) was used to investigate the structure of EmrE in the SIMPLEx format. This technique allows characterization of biomolecular structures in solution that can be used to formulate working models^34^. Given the small size of OspA (∼90 amino acids) and the fact that OspA-EmrE-ApoAI* appeared roughly spherical in negative staining images (Supplementary Fig. 7), we anticipated that it might be masked in the molecular envelopes, resulting in poor data interpretation and model building. To circumvent this issue, we examined the highly soluble and larger ΔspMBP in the N-terminal position of the SIMPLEx chimera. Monodisperse ΔspMBP-EmrE-ApoAI* fusions were prepared as dimers or tetramers using Ni^2+^-affinity chromatography and SEC in the absence of detergents (Supplementary Fig. 2c). SAXS profiles of dimeric and tetrameric ΔspMBP-EmrE-ApoAI* were obtained at a total protein concentration of 1 mg ml^−1^ (Supplementary Fig. 9a). The extrapolated SAXS intensity at zero angle, *I*(0), was proportional to molecular mass and showed the expected factor of two increase from the dimer to the tetramer samples. Differences in the size and shape of dimers and tetramers were revealed by comparing radii of gyration and pair distance distribution functions, respectively (Supplementary Fig. 9b,c). The absence of aggregation in both dimeric and tetrameric forms of ΔspMBP-EmrE-ApoAI* was confirmed by an unchanging radius of gyration as the protein concentration was increased by more than order of magnitude, to 10 mg ml^−1^ (Supplementary Fig. 9c).

**Figure 4:**
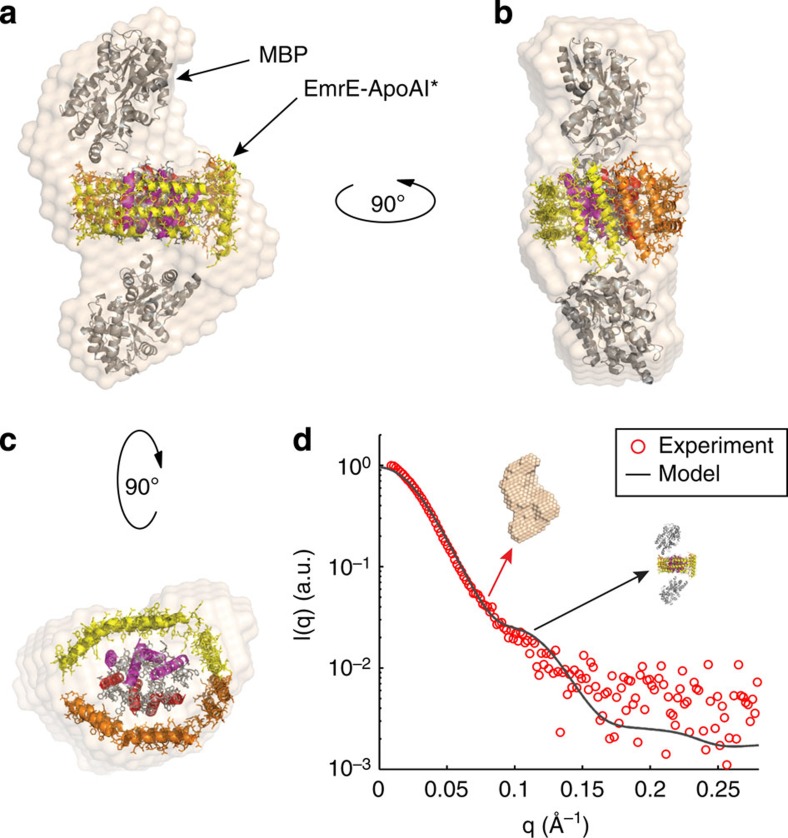
Structural characterization of ΔspMBP-EmrE-ApoAI* by SAXS. (**a**–**c**) Multiple views of the reconstructed particle envelope calculated *ab initio* from the dimer SAXS data (red circles in **d**) using DAMMIF^35^. The structural model with the lowest *χ*^2^-value is docked into the envelope using SUPCOMB. The images represent (**a**) side view, (**b**) lateral view, rotated −90° around *z* axis from view in (**a**) and (**c**) top view, rotated −90° around *y* axis from view in (**a**). ΔspMBP proteins were removed from the representation in (**c**) to enhance the visualization of the modelled interactions between EmrE and ApoAI*. The model was constructed using ΔspMBP crystal structure (pdb ID: 1NL5), ApoAI lipid-free crystal structure (pdb ID: 2A01) and electron microscopy-derived structure of dimeric EmrE (pdb ID: 2I68). EOM analysis provided the structural framework for this model. (**d**) Comparison between the experimental scattering profile of the dimer (red circles) and the theoretical profile calculated for the proposed model using CRYSOL (solid line). Goodness of fit is accessed by a *χ*^2^-test. A *χ*^2^-value of 0.174 indicates that the calculated SAXS curve agrees with the experiment data within error, consistent with the good alignment observed between the proposed model and the reconstruction in (**a**–**c**).

Reconstructions of the molecular envelope of dimeric ΔspMBP-EmrE-ApoAI* were computed *ab initio* using DAMMIF software^35^. The average of 20 bead models is shown in Fig. 4. No symmetry was imposed in the reconstruction algorithm. All 20 models are similar (mean normalized spatial discrepancy (NSD)=0.636). Attempts to dock the known structures of the individual proteins into the envelope suggests an antiparallel orientation of the two monomeric ΔspMBP-EmrE-ApoAI* units. Further evidence for this configuration of proteins in the complex arose from direct fitting of the experimental SAXS curve (*I*(*q*) versus *q*) using an Ensemble Optimization Method (EOM)^36^. Ten thousand potential models of the ΔspMBP-EmrE-ApoAI* dimers were built from known rigid crystal structures of the three protein domains and randomly generated flexible domain linkers, resulting in a variety of possible orientations. In addition, as suggested by computational models^37^, we assigned flexible regions to the continuous helical domains observed in the ApoAI monomer structure (pdb ID: 2A01; Supplementary Fig. 10). A genetic algorithm was used by EOM to select ensembles of conformations from the large pool, whose averaged theoretical scattering profile best fit the SAXS data (Supplementary Fig. 9d). The final optimized ensemble of dimer models consisted predominantly of two conformations, which, interestingly, possessed similar quaternary configurations (Supplementary Fig. 9d). The EOM models demonstrated that ΔspMBP proteins were on the opposite sides of the dimeric particle. When the two models were aligned together using SUPCOMB^38^ (Supplementary Fig. 9d), we found that the two ApoAI* proteins tended to wrap around the EmrE dimer, consistent with the evidence of solubility observed *in vivo* and in detergent-free solutions. Finally, these selections were consistent with the structures that docked into the reconstructed envelope.

The lipid-binding domain of ApoAI (residues 44–243) consists of a series of eight 22-mer and two 11-mer amphipathic α-helices, which are interrupted by prolines or glycines^39^. To take into account both the high strand flexibility in the regions between the 10 helices as well as the hydrophobic shielding nature of ApoAI* against EmrE, we constructed several alternative models based on the structural framework provided by EOM. These models were compared to experimental data using CRYSOL to compute their SAXS profiles^40^. The *χ*^2^-value was also computed and used to assess goodness of fit. We docked the model with the lowest *χ*^2^-value 
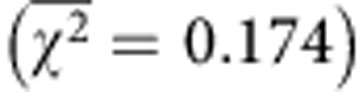
 into the reconstructed envelope (Fig. 4a–c). On the basis of these results, we hypothesize that ApoAI* folds perpendicularly to the EmrE helices in a manner that resembles a shield (Fig. 4c). This requires long helices (beyond 22-mer) that resemble those of the lipid-free ApoAI protein structure^41^ (Supplementary Fig. 10). In such a conformation, the last two helices of ApoAI form a small domain that is independent of the main helical bundle. The linker between these two domains is a pair of glycine residues (Gly185, Gly186) that provide extreme flexibility. Once the shield has been built, there is still significant hydrophobicity exposed to water where the dimers meet laterally. We predict that this small C-terminal domain shields this region, standing parallel to the EmrE helices. According to this shield model, protein–protein interactions between ApoAI* and EmrE promote solubilization of the IMP by shielding the ‘hydrophobic core' of the fusion protein from water without compromising the IMP's ability to form homo-oligomers or bind ligands.

## Discussion

Here we report a new strategy for the solubilization of IMPs based on the affinity for hydrophobic surfaces displayed by amphipathic proteins. This affinity was exploited to create specific protein–protein interactions *in vivo* between an amphipathic protein and a given IMP. Specifically, a truncated version of human ApoAI, ApoAI*, fused to the C terminus of an array of structurally diverse IMPs resulted in efficient *in vivo* solubilization of the IMP target without the need for detergents or lipid reconstitutions. By directing the expression of IMPs to the cytoplasm of *E. coli*, we take advantage of this compartment's ability to support recombinant product yields exceeding 50% of the total cellular protein^42^ while eliminating the energy intensive process of membrane integration. The end result is the accumulation of non-aggregated, water-soluble IMPs at high titres (∼5–10 mg l^−1^ of culture).

The yield of IMPs achieved with SIMPLEx compares favourably to previous efforts describing the production of various IMPs that were tested here. For example, ∼1 mg l^−1^ of EmrE was obtained using membrane-targeted expression in *E coli*^43^. To produce this much EmrE required a complicated multi-step procedure involving chloroform/methanol solvent mixtures to extract EmrE followed by solubilization in SDS detergent. Moreover, as is typical for many membrane protein expression campaigns, more than 50 different variables related to culture conditions, solvent mixture ratios and detergent choices had to be screened to identify the optimal production conditions. In contrast, *in vivo* solubilization using SIMPLEx yielded greater amounts of EmrE in a process that involved only standard expression and purification conditions. In the case of recombinant expression of cyt *b*_5_ in *E. coli*, a number of strategies have been investigated. These include: (1) cytoplasmic expression of truncated cyt *b*_5_ comprised of just the soluble, haeme-containing domain; (2) periplasmic expression of the same soluble domain; and (3) membrane-targeted expression of the full-length cyt *b*_5_. When the soluble cyt *b*_5_ domain was expressed in the cytoplasm or periplasm, yields of ∼5 mg l^−1^ soluble protein were obtained^44^,^45^. However, despite the generation of mature, haeme-assembled cyt *b*_5_ in soluble form, these truncated variants were incapable of stimulating 17,20-lyase activity^33^. Alternatively, full-length cyt *b*_5_ has been expressed in *E. coli* membranes with a similar yield of ∼5 mg l^−1^ of cell culture following extraction with acetonitrile and purification^46^. Unlike the truncated variants, full-length, detergent-solubilized cyt *b*_5_ stimulated 17,20-lyase activity^33^. By way of comparison, SIMPLEx yielded 5–8 mg l^−1^ of full-length, haeme-assembled cyt *b*_5_ in a soluble conformation that stimulated lyase activity on par with the detergent-solubilized protein but without the need for detergents. Taken together, these comparisons highlight the ability of SIMPLEx to yield competitive quantities of different IMP targets using a facile, cost-effective procedure that has the potential to be transferred widely to other targets.

In the specific case of EmrE, the IMP was rendered highly soluble in the absence of detergents, exhibiting characteristics of globular proteins while retaining the IMP fold as judged by its near-native ligand-binding dissociation constants. Moreover, the ApoAI*-solubilized EmrE was amenable to structural characterization including negative staining electron microscopy, dynamic light scattering and SAXS data collection. The SAXS analysis shed light on the structural plasticity that enables ApoAI* to form an amphipathic shield for sequestering IMPs from water and promoting their solubilization. Whether the flexibility of ApoAI* will present a challenge for crystallization trials is currently under investigation. It should be pointed out, however, that ApoAI itself has been crystallized^41^. Nonetheless, if flexibility proves to be an issue, it is possible to overcome this by further engineering the decoy protein, the linker length and composition, and the amphipathic protein (or use a completely different amphipathic protein altogether, which we have successfully done in related unpublished studies). The good news is that alternative methods for structure determination such as SAXS and three-dimensional reconstruction with electron microscopy, which is advancing towards atomic resolution, are compatible with our method. Overall, by providing high yields, proper folding, and preserved activity of target proteins, our technique represents a powerful new addition to the toolkit for high-throughput and structural studies of IMPs of varying size and topology.

## Methods

### Bacterial strains and growth conditions

*E. coli* strain DH5α was used for cloning while protein expression was carried out in *E coli* strain BL21(DE3). Overnight cultures were diluted 1:500 in terrific broth supplemented with the appropriate antibiotic (50 μg ml^−1^ kanamycin or 100 μg ml^−1^ ampicillin) and grown at 30 °C until culture optical density (OD_600_) reached ∼1.0. The temperature was then lowered to 16 °C and protein expression was induced with isopropyl-β-D-thiogalactoside to a final concentration of 0.1 mM. Cells were collected 20 h post induction.

### Plasmid construction

The basic construct in these studies was a tripartite fusion between a soluble cytoplasmic decoy protein, a target IMP and N-terminally truncated human ApoAI (Δ1–43; ApoAI*) in pET28a (Novagen). For the decoy, the N-terminal domain (residues 4–93) of the engineered OspA A (OspA) of *Borrelia burgdorferi*^47^ was prepared by PCR and subcloned in pET28a between the NcoI and NdeI restriction sites. This cloning resulted in an additional Gly placed immediately after Met4. As an alternative decoy for cytoplasmic expression, *E. coli* MBP lacking its N-terminal signal peptide (ΔspMBP) was used. The gene encoding ΔspMBP (residues 27–368) was subcloned from plasmid pMALc2x (New England Biolabs) in pET28a using the same restriction sites as above. This cloning resulted in an additional Gly residue immediately before Lys27 of ΔspMBP. A reverse primer introduced a triple Ala motif following Asn368 in ΔspMBP and before the NdeI site. Subsequently, ApoAI* (residues 44–243) was subcloned between EcoRI and NotI sites of the pET28a plasmid containing either OspA or ΔspMBP. The final plasmids were arranged as follows: (NcoI)-decoy protein-(NdeI)-IMP-(EcoRI)-ApoAI*-(NotI)-6 × His. All IMP targets were subcloned between NdeI and EcoRI. In the absence of an IMP target, this plasmid served as the OspA-ApoAI* control. Additional control plasmids for expressing OspA-IMP/ΔspMBP-IMP (lacking ApoAI*) or unfused IMPs (lacking OspA/ΔspMBP and ApoAI*) were constructed similarly. Plasmids for fluorescence microscopy were created by introducing full-length GFP to the C terminus of the different chimeras described above. This cloning involved ligating the gene encoding GFP between NotI and XhoI sites in each of the different pET28a plasmids described above. Plasmids for protein expression via the Tat pathway were created in plasmid pTrc99A (Pharmacia). In brief, overlap extension PCR was used to join DNA encoding the Tat-dependent spTorA signal peptide to the 5′ end of DNA encoding EmrE, OspA-EmrE, OspA-ApoAI* or OspA-EmrE-ApoAI*. During overlap extension PCR, NcoI and XbaI sites were introduced at the 5′ and 3′ ends, respectively, and a 6 × -His tag was also introduced at the 3′ end of all constructs. The resulting overlap extension PCR products were ligated into the corresponding sites in pTrc99A. All plasmids were confirmed by DNA sequencing at the Cornell Biotechnology Resource Center.

### Subcellular fractionation

Following protein expression, 20 ml of cells expressing IMP fusions were harvested. Cultures were normalized by OD_600_ and culture aliquots were pelleted via centrifugation for 10 min at 4 °C and 4,000 *g*. Cells were then resuspended in lysis buffer containing 30 mM Tris pH 8.0, 500 mM NaCl and 40 mM imidazole pH 8.0 and lysed using a homogenizer (Avestin Emulsiflex C5). To separate soluble proteins from membranes, the homogenate was ultracentrifuged (100,000 *g*) for 1 h at 4 °C and the supernatant was collected as the soluble fraction. Detergent soluble fractions were obtained by treating the pellets resulting from the previous step with 10 ml of lysis buffer containing 2% *n*-dodecyl-β-D-maltoside (DDM; Anatrace). Pellets were resuspended by douncing. Partitioning of membrane proteins into the DDM-containing lysis buffer was achieved by rotating the lysate at 4 °C for 2 h. Following ultracentrifugation (100,000 *g*) for 1 h at 4 °C, the supernatant represented the ‘detergent-solubilized' fraction and the pellet represented the ‘insoluble fraction.' For experiments that involved the isolation of periplasmic fractions, cells were initially resuspended in 20% sucrose, 30 mM Tris-HCl pH 8.5, 1 mM EDTA and 1 g l^−1^ lysozyme and incubated at room temperature for 10 min. Following centrifugation (10 min at room temperature and 10,000 *g*), cell pellets were fractionated according to standard ice-cold osmotic shock. The supernatant resulting from the centrifugation step (10 min at 4 °C and 15,000 *g*) was taken as the periplasmic fraction, while the remaining pellet was used to prepare the soluble cytoplasmic fraction as described above. IMPs in the various fractions were separated by SDS–polyacrylamide gel electrophoresis using 10% polyacrylamide gels (Bio-Rad) and subsequently detected by Western blotting according to standard protocols using a 1:5,000-diluted monoclonal anti-6 × -His HRP-conjugated antibody (Abcam).

### Protein purification

Proteins were purified from soluble fractions in one of two ways. For chimeras containing OspA, the supernatant containing the 6 × -His-tagged protein of interest was purified using an ÄKTA Explorer FPLC system (GE Healthcare) over a Ni^2+^ Sepharose High-Performance HisTrap HP column (GE Healthcare). For chimeras containing ΔspMBP, purification was performed according to the manufacturer's protocol supplied with pMAL vectors (New England Biolabs). SEC was performed on all 6 × -His-tagged and ΔspMBP-tagged purified proteins. Standards used to calibrate the SEC column were a lyophilized mix of thyroglobulin, bovine γ-globulin, chicken ovalbumin, equine myoglobin and vitamin B12, MW 1,350-670,000, pI 4.5-6.9 (Bio-Rad). Proteins were stored at a final concentration of 1 mg ml^−1^ in SEC buffer (20 mM Tris pH 7.5, 50 mM NaCl, 1 mM EDTA pH 8.0) at 4 °C. Expression and purification of EmrE was according to standard protocols^43^.

### Lipid content

Analytical measurement of lipid content for SIMPLEx solubilized EmrE and cyt *b*_5_ was performed by acid digestion of the organic sample to produce inorganic phosphate. Subsequently, total phosphorus was measured according to standard protocols^48^.

### Ligand binding

Fluorescence spectra of protein samples were collected using a Fluorolog-Tau-3 time-resolved spectrofluorometer (Horiba). Protein concentration of OspA-EmrE-ApoAI* in SEC buffer and EmrE in DDM-containing buffer (20 mM Tris-HCl, pH 7.5, 150 mM NaCl and 0.08% w/v DDM) was 10 μM. Fluorescence spectra using a 295-nm excitation were collected after each volume of ligand. A small magnetic stir bar was added to the 1-cm path-length quartz cuvette containing either the sample or buffer. The stirring speed was set such that the surface was not noticeably disturbed. Approximately 1 min was allowed between the addition of ligand and the beginning of spectra collection. A total of three replicates were performed with the first sample preparation to control for experimental variability. After this, only one experimental replicate of the following second and third sample preparations were performed. Thus, each ligand-binding curve reflects the average of three replicates from three different protein preparations to control for biological and preparation variability. A 10-nm slit width was used for both excitation and emission. The interval was set at 2.0 nm and the integration time at 0.1 s. Only one scan of the emission between 300 and 400 nm was collected per titration. The 295-nm excitation was used to select for the tryptophans in the EmrE samples. Samples were diluted and assayed in a 1-cm quartz cuvette at room temperature. A blank titration of SEC and DDM buffer alone without EmrE was performed to observe the baseline signal. All ligands were titrated to near saturation based on a ligand concentration where further titrations resulted in little to no observable change in the fluorescence intensity. Quenching of fluorescence was recorded and plotted using GraphPad PRISM 6.

### Lyase assays

In a 2-ml polypropylene tube, microsomes containing native CYP17A1 (5 pmol) and cytochrome P450 oxidoreductase (POR) from transformed yeast were preincubated with haeme-titrated cyt *b*_5_ variants (5–20 pmol), at room temperature for 5 min before adding substrate. The reaction mixture was then diluted to 0.2 ml with 50 mM potassium phosphate buffer (pH 7.4) and substrate 17-hydroxypregnelonone (17-P5; 5 μM with 80,000 c.p.m. in methanol, 2% of incubation volume) was added. The resulting mixture was preincubated at 37 °C for 3 min before adding NADPH (1 mM) and incubating at 37 °C for another 20 min. The reaction mixture was extracted with 1 ml dichloromethane, and the organic phase was dried under nitrogen flow. Steroids were analysed using an Agilent 1260 Infinity high-performance liquid chromatography system with ultraviolet detector and β-RAM4 in-line scintillation counter (LabLogic, Brandon, FL). Extracted steroid products were dissolved in 20 μl of methanol, and 5 μl injections were resolved with a 50 × 2.1 mm, 2.6 μm, C8 Kinetex column (Phenomenex, Torrance, CA), equipped with a guard column at a flow rate of 0.4 ml min^−1^. A methanol/water linear gradient was used: 27% methanol from 0 to 0.5 min, 39% to 16 min, 44% to 20 min, 60% to 22 min, 71% to 30 min, 75% to 30.5 min, 27% to 33 min. Products were identified by retention times of external standards chromatographed at the beginning and ends of the experiments. The flow rate of the scintillation cocktail (Bio-SafeII, Research Products International, Mount Prospect, IL) was 1.2 ml min^−1^ and the data were processed with Laura4 software (LabLogic).

### Spectroscopic analysis of cyt *b*_5_ redox state

The absorbance at 409 nm (Abs_409_) of the oxidized cyt *b*_5_ constructs (1 nmol) in 0.2 M potassium phosphate, pH 7.5, with 0.05% CHAPS in a final volume of 0.3 ml was monitored for 2.5 min at 25 °C with data points collected every 5 s using a Shimadzu 2600 ultraviolet–visible spectrophotometer (Addison, IL). For the reduction of cyt *b*_5_, POR (32 pmol) was incubated with cyt *b*_5_ variants (1 nmol) and 1.1 mM NADPH in 0.2 M potassium phosphate, pH 7.5, with 0.05% CHAPS in a final volume of 0.3 ml. The absorbance at 424 nm (Abs_424_) was monitored as described above. Analysis included the superimposition of oxidized and reduced spectra.

### Confocal microscopy

*E. coli* expressing proteins with C-terminally fused GFP were harvested and diluted 1:100 in Luria-Bertani medium. Poly-lysine (Sigma)-coated slide glass was used to mount the cells. A cover glass was placed over the cells and sealed in place with clear nail polish. Cells were imaged within 1 h of their preparation with a Zeiss LSM710 confocal microscope equipped with a × 100 oil immersion objective.

### Dynamic light scattering

Freshly purified OspA-EmrE-ApoAI* (2 μM) in 20 mM Tris 7.5, 50 mM NaCl, 5% glycerol, 1 mM EDTA pH 8.0 was equilibrated for 3 min in a sealed 15-μl quartz cuvette at 20 °C before recording with Dynapro Dynamics Light Scattering (Protein Solutions). A total of 30 scattering intensity acquisitions were recorded for each sample tested (10 acquisitions of 1 s per measurement). Data were processed using Dynamics Dynapro Control Software v.6.3.40.

### Negative staining electron microscopy

Freshly purified OspA-EmrE-ApoAI* was prepared at different concentrations (0.5, 0.25, 0.1 and 0.05 mg ml^−1^) for negative staining by applying a 5-μl protein drop to a carbon-coated grid (300-mesh copper grid) for 2 min and blotting with filter paper to remove excess solution. A second solution of 1.5% uranyl acetate was immediately applied for another 2 min. Dried grids were examined using a FEI Tecnai 12 Spirit Twin electron microscope. Twenty fields for each sample concentration were randomly photographed at different magnification levels and later analysed with ImageJ software.

### SAXS

SAXS data were collected at the Cornell High Energy Synchrotron Source (CHESS) G1 station in Ithaca, New York. Protein samples of ΔspMBP-EmrE-ApoAI* were exposed with a 250 × 250 μm beam of 9.968 keV X-ray. Sample preparation included centrifugation at 30,000*g* for 30 min and filtration to remove any aggregates. Samples (30 μl) were loaded and oscillated in the beam using an automated system with a plastic chip-based sample cell (2-mm path) and polystyrene X-ray transparent windows. The sample cell and X-ray flight path were placed under vacuum to reduce background scattering. Scattering patterns were captured on a Pilatus 100K-S detector (Dectris, Baden, Switzerland) at 1,504-mm distance. The exposure time was 5 s for each image and 10 images were recorded for each sample. All mathematical manipulations of the data (azimuthal integration, normalization, averaging and buffer subtraction) as well as error propagation were carried out using RAW software^49^. The range of momentum transfer was calculated to be 0.0068<q=4*π* sin(*θ*)/*λ*<0.28 Å^−1^, where 2*θ* is the scattering angle and *λ*=1.257 Å is the X-ray wavelength. Dimer and tetramer samples were run at a range of concentrations (0.3, 0.6, 1.0, 2.0, 5.0, and 10 mg ml^−1^) to evaluate for possible concentration effects. Molecular weight estimated from a lysozyme standard (3.5 mg ml^−1^, 50 mM NaOAc, 50 mM NaCl pH 4.0) agreed with our expectations within error. Radius of gyration (*R*g) was calculated using both Guinier approximation^50^ and the inverse Fourier transform method as implemented in the GNOM-ATSAS 2.3 package by D. Svergun EMBL-Hamburg. The pair distance distribution function P(r) was calculated using the GNOM program^51^. The maximum dimension of the particle, *D*_max_, was estimated based on the goodness of the data fit and smoothness of the decaying tail. The GNOM output file for the dimer was used as input to DAMMIF^35^ to perform *ab initio* shape reconstruction without imposing any symmetry. The 20 reconstructed bead models were superimposed and averaged using DAMAVER in the automatic mode. The mean NSD was 0.636±0.047 (*n=*20), where an NSD value <1 indicates close agreement between different reconstructed models.

### EOM and structural model refinement

EOM^36^ was used to model the flexible linkers between the three protein domains and construct possible ΔspMBP-EmrE-ApoAI* dimer models from five components: one EmrE dimer (Cryo-EM model, pdb ID: 2I68), two ApoAI monomers (full-length ApoAI, pdb ID: 2A01 or lipid-bound ApoAI, pdb ID 3K2S or ApoAI*, pdb ID 1AV1) and two MBP monomers (pdb ID: 1NL5). During test runs, we found that dimer models containing the extended conformation of ApoAI (pdb ID: 3K2S and 1AV1) fit the experimental data poorly due to the large disagreement between the size of the models (average Rg=75 Å) and the measured Rg (49.85±0.99 Å, where *n=*4 and error is defined as s.d.). Thus, only the compact ApoAI conformation (pdb ID: 2A01) was used for further EOM analysis. For each EOM run, 10,000 structural models are first generated. EOM then uses a genetic algorithm to select from this pool of models, an ensemble of dimer conformations, whose combined theoretical scattering intensity best describes the experimental SAXS data of the dimer. A *q* range of 0.009–0.28 Å^−1^ was used for EOM fitting. An optimized ensemble was first generated from a pool composed of half symmetric and half asymmetric models (for symmetric models, P2 symmetry was imposed) and was found to be populated with mostly symmetric models. We also found that the overall EOM fitting assessed by (*χ*^2^) values was improved when rotational freedom was allowed for the flexible GGA linker between the main bundle and the C-terminal domain of ApoAI* (Supplementary Fig. 9), indicative of the conformational change of ApoAI* on EmrE binding. Hence, we refined our sampling pool to contain only symmetric models with free GGA linkers in ApoAI*, and a new process of ensemble optimization was conducted. The final optimized ensemble contains only two most populated conformations with similar configuration of the five components, consistent with the monodispersity observed in SAXS reconstruction of the dimer. Owing to high computational cost, EOM analysis was used mainly to model interdomain interactions. To further refine the in-solution structure of the ΔspMBP-EmrE-ApoAI* dimer, especially to compare the conformational variants of the highly flexible ApoAI* protein on binding to EmrE, we built several hypothetical models based on biochemical evidence and the structural frame provided by EOM. Agreement between the experimental data and these potential structural models was assessed by evaluating the following chi-square:


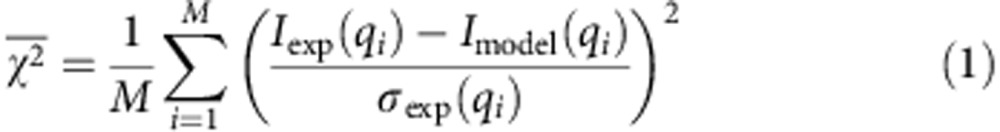


where *I*_exp_(q_i_) is the experimental scattering intensity at q_i_, *I*_model_ (q_i_ ) is the scattering intensity calculated from models using CRYSOL^40^, *σ*_exp_(q_i_) is the experimental error and *M* is the number of data points in q space. A *q* range of 0.009–0.28 Å^−1^ was also used for the fitting. The best fit revealed by the minimal *χ*^2^-value (
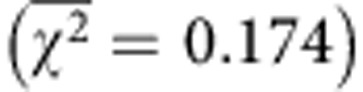
) is shown in Fig. 4.

## Author contributions

D.M., Y.C. and R.J.T. designed and performed the research, analysed the data. D.M. wrote the paper. J.L. and H.-M.P. performed research. A.K. designed research. L.P. designed research and analysed the data. R.J.A. designed research and analysed data. M.P.D. designed the research, analysed the data and wrote the paper.

## Additional information

**How to cite this article:** Mizrachi, D. *et al.* Making water-soluble integral membrane proteins *in vivo* using an amphipathic protein fusion strategy. *Nat. Commun.* 6:6826 doi: 10.1038/ncomms7826 (2015).

## Supplementary Material

Supplementary Information Supplementary Figures 1-10
