# Supplementary material for: Making water-soluble integral membrane proteins in vivo using an amphipathic protein fusion strategy
Source: Nat Commun. 2015 Apr 8;6:6826. doi: 10.1038/ncomms7826 (PMC4403311; doi:10.1038/ncomms7826)
Supplement: Supplementary Information — Supplementary Figures 1-10 [file ncomms7826-s1.pdf]

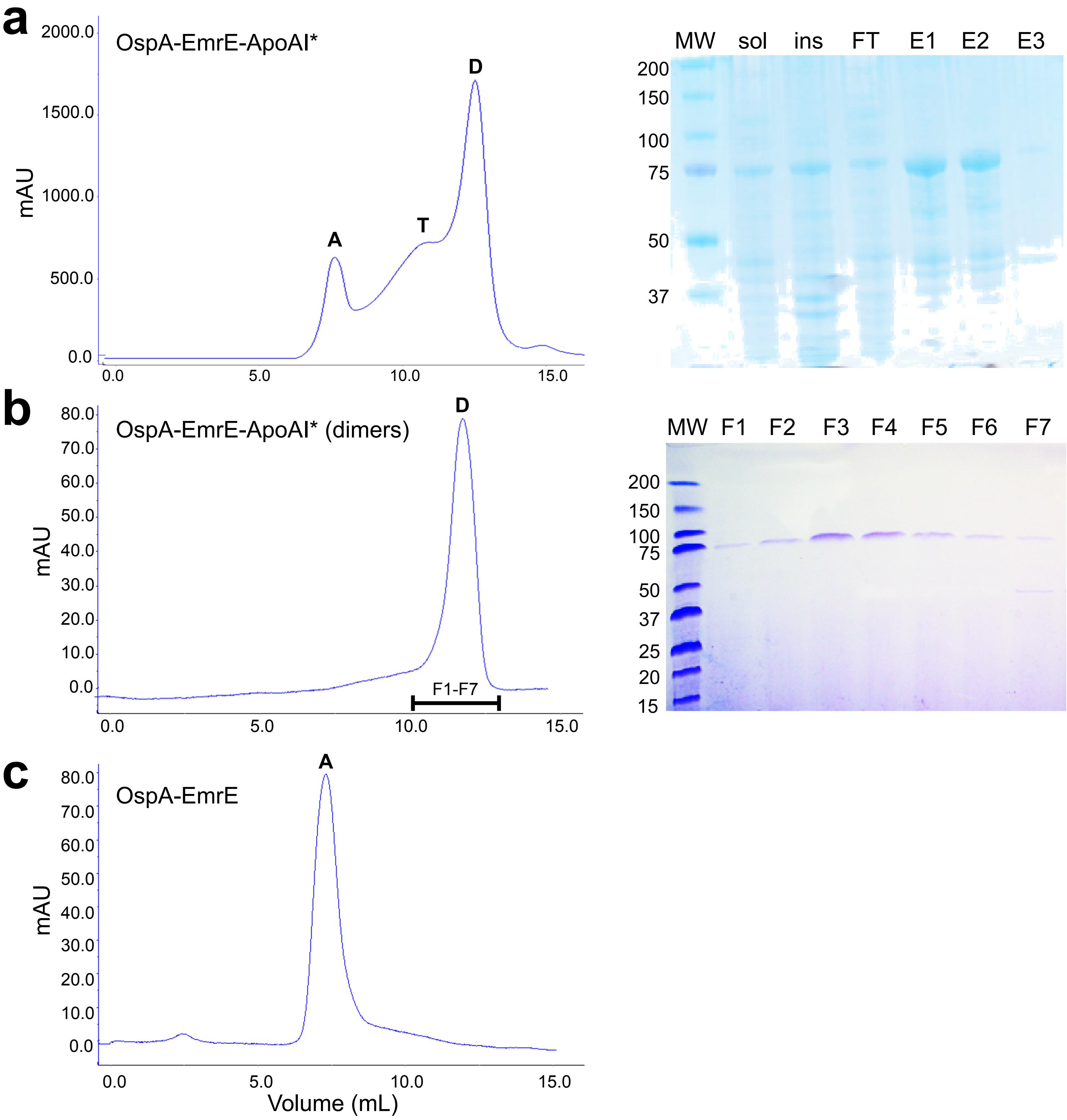

Supplementary Figure 1. Size exclusion chromatography of EmrE constructs. (a) SEC profile of Ni^{2+}-affinity chromatography purified OspA-EmrE-ApoAI* over Superdex 200 10/300 GL (GE Healthcare). The distinguishable species are labeled as aggregates (A), tetramers (T) and dimers (D). The Coomassie-stained gel showing soluble (sol), insoluble (ins) and flow-through (FT) fractions as well as elution fractions (E1-E3) from Ni^{2+}-affinity column. Molecular weight (MW) ladder is shown on the left. (b) Pooled dimer fractions containing dimers of OspA-EmrE-ApoAI* reapplied to the Superdex 200 column. The profile indicates that dimers in solution remain homogeneous and monodisperse. Bar indicates SEC fractions F1-F7 collected for SDS-PAGE analysis. Coomassie-stained gel showing SEC fractions F1-F7 as indicated. Molecular weight (MW) ladder is shown on the left. (c) SEC profile of Ni^{2+}-affinity chromatography purified OspA-EmrE over Superdex 200 10/300 GL (GE Healthcare). OspA-EmrE elutes exclusively as an aggregated protein.

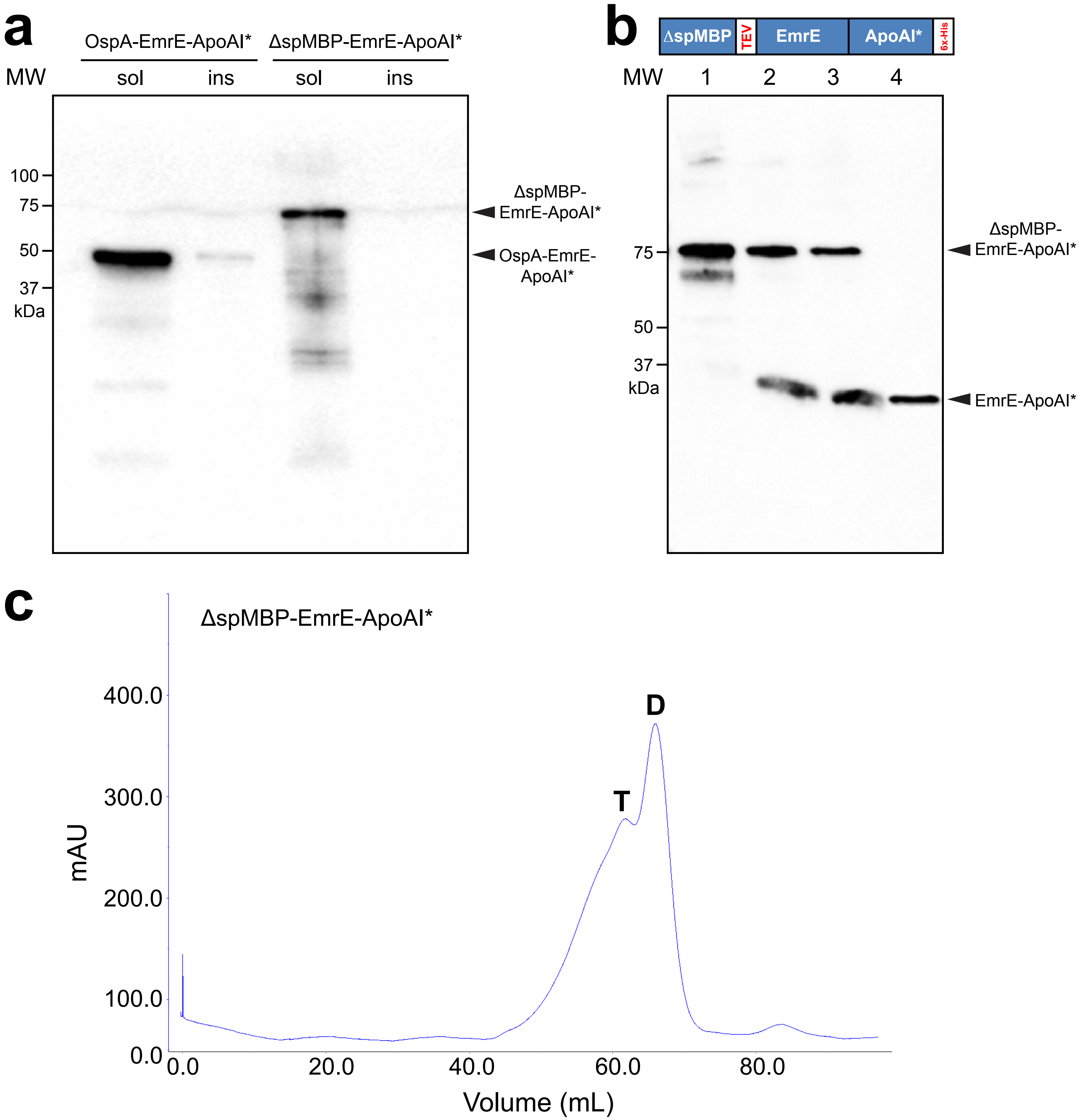

Supplementary Figure 2. Use of ΔspMBP as an alternative soluble "decoy" protein. (a) Western blot analysis of soluble (sol) and insoluble (ins) fractions prepared from *E. coli* strain BL21(DE3) expressing either OspA-EmrE-ApoAI* or ΔspMBP-EmrE-ApoAI* as indicated. (b) Western blot analysis of purified ΔspMBP-TEV-EmrE-ApoAI* that was (1) untreated, (2) treated with TEV protease in solution, (3) treated with TEV on amylose column, then eluted, and pulled down with Ni^{2+}-affinity column; or (4) treated with TEV on amylose column and collected as flow-through. (c) SEC profile of amylose chromatography-purified ΔspMBP-EmrE-ApoAI* over Superdex 200 pg 16/600 (GE Healthcare). The distinguishable species are labeled as tetramers (T) and dimers (D).

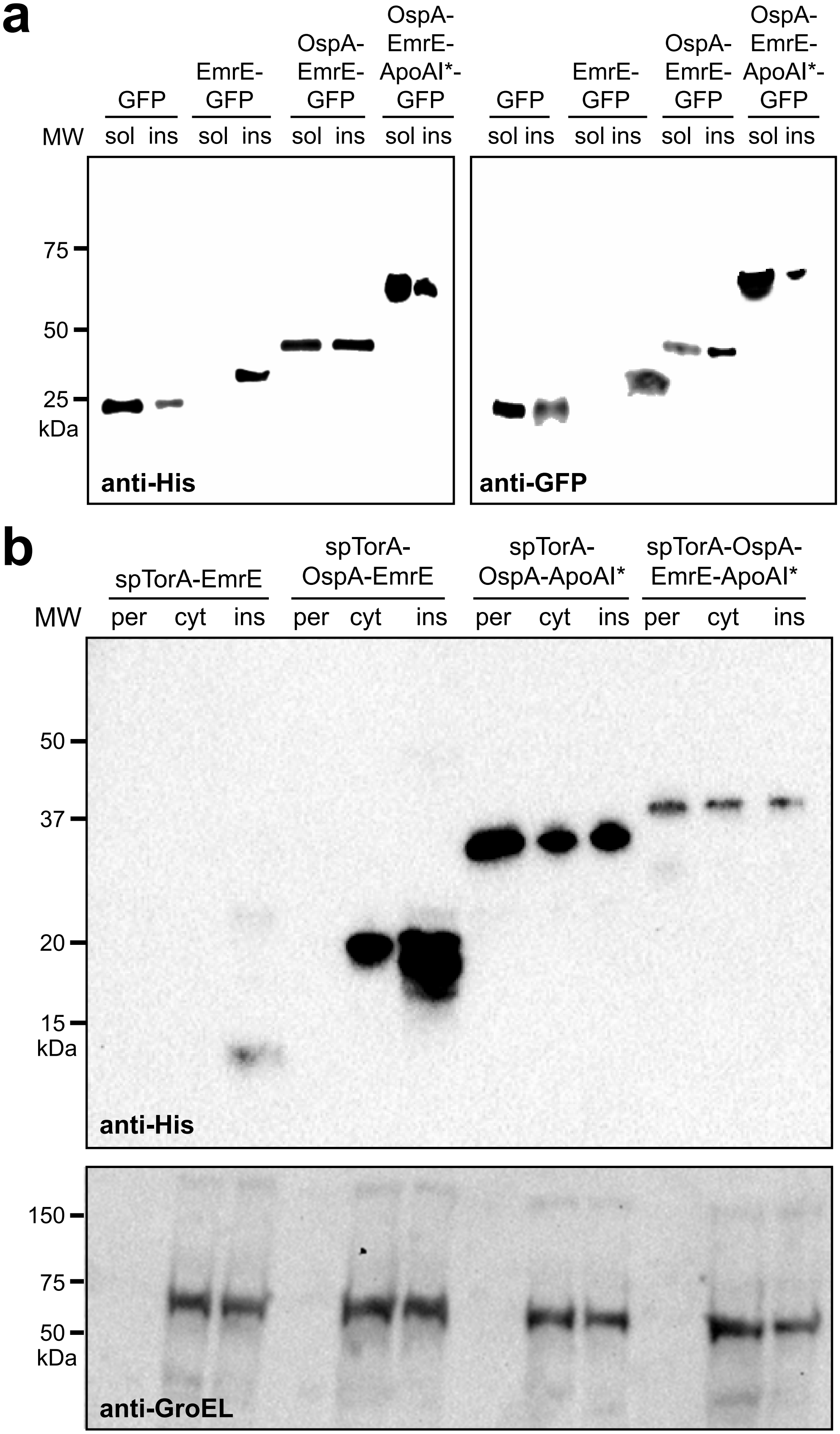

Supplementary Figure 3. Subcellular accumulation of GFP- and spTorA-tagged EmrE. (a) Western blot analysis of soluble (sol) and insoluble (ins) fractions prepared from *E. coli* strain BL21(DE3) expressing GFP-tagged constructs as indicated. Fusions were detected with anti-His (left panel) or anti-GFP (right panel) antibodies. (b) Western blot analysis of periplasmic (per), cytoplasmic (cyt), and insoluble (ins) fractions prepared from *E. coli* strain BL21(DE3) expressing either spTorA-EmrE, spTorA-OspA-EmrE, spTorA-OspA-ApoAI*, or spTorA-OspA-EmrE-ApoAI* as indicated. Different fusions were detected with anti-His antibody (top panel) while quality of fractionations was verified by probing with anti-GroEL antibody (bottom panel). Molecular weight (MW) markers are shown on the left.

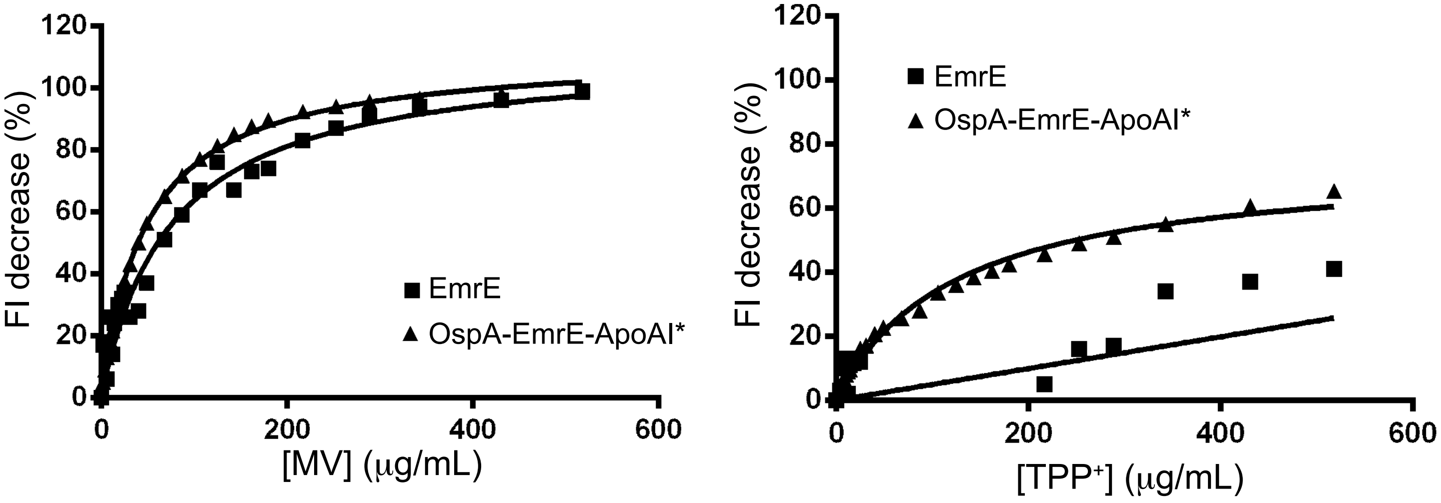

Supplementary Figure 4. Ligand-binding activity of solubilized EmrE. Activity of dimeric, detergent-free OspA-EmrE-ApoAI* or organic-extracted detergent-solubilized EmrE, both of which were purified from BL21(DE3) cells. Activity ssays were performed with methyl viologen (MV), or tetraphenylphosphonium (TPP^+) as substrates. Data is expressed as the mean of biological triplicates where the standard error of the mean (SEM) was <5%.

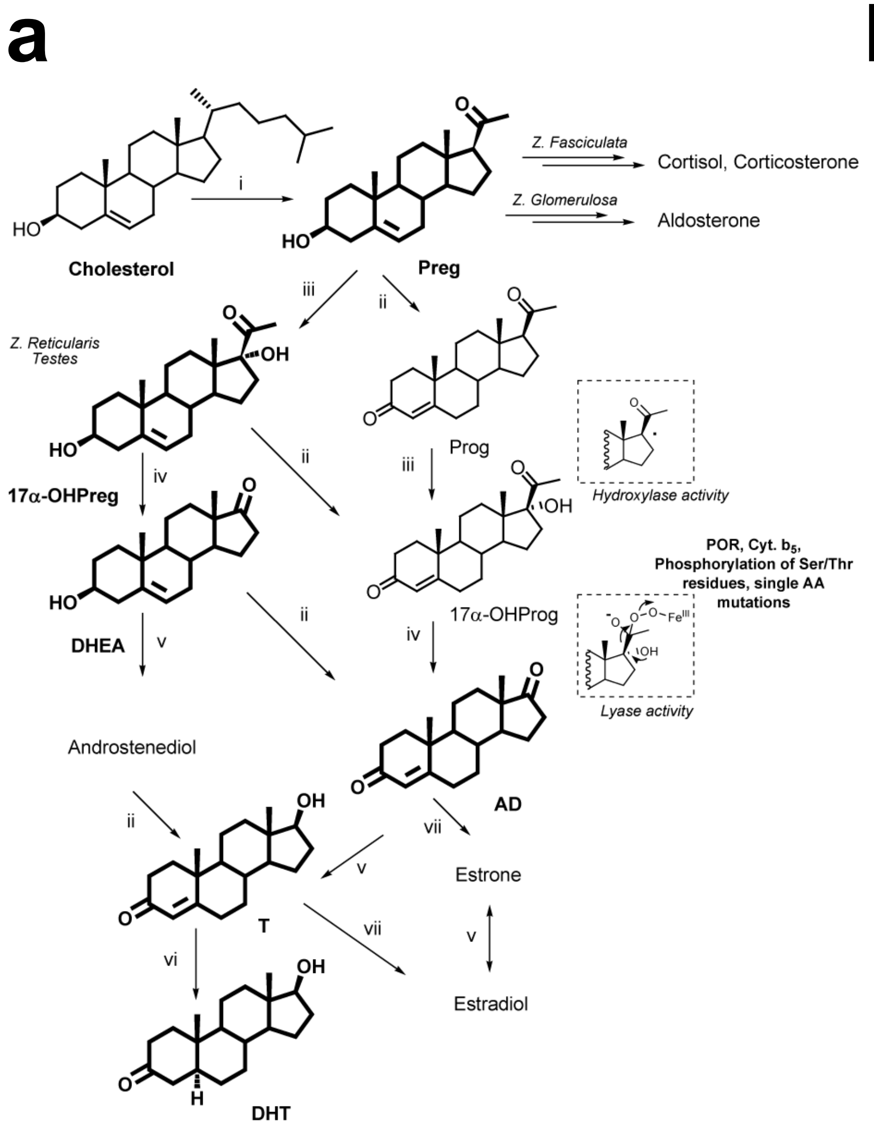

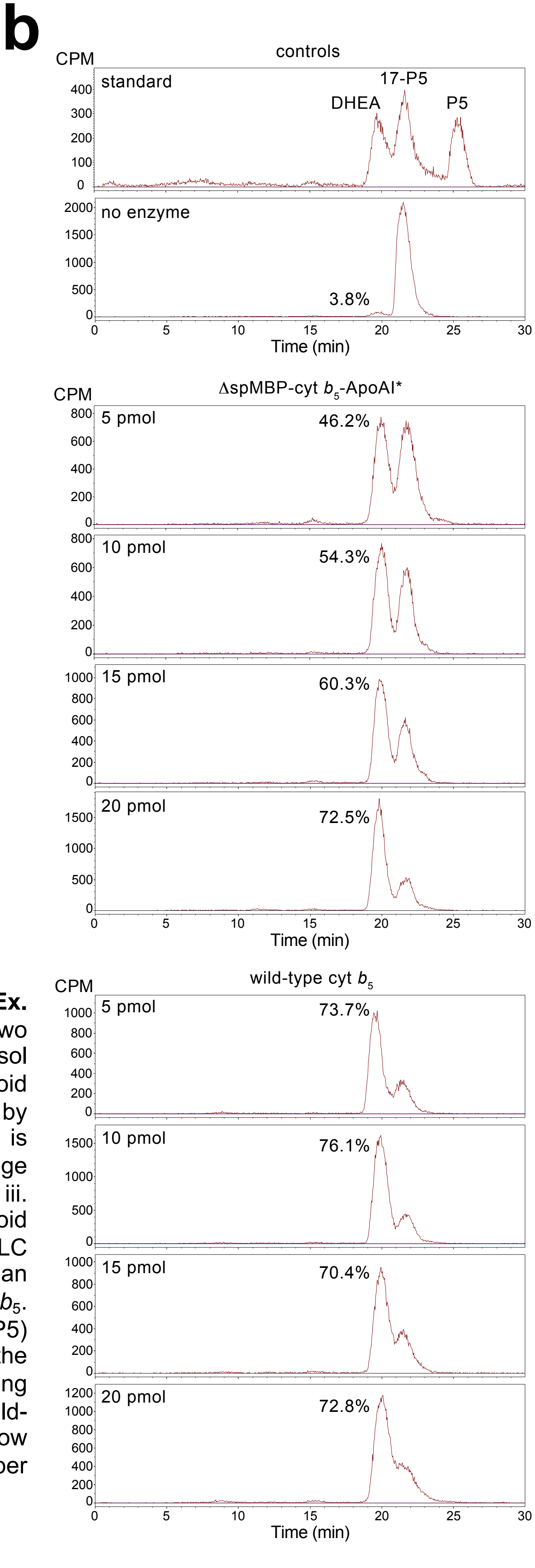

Supplementary Figure 5. Solubilization of cyt b_5 using SIMPLEx. (a) Schematic of the steroidogenesis pathway. CYP17A1 has two distinct activities: 17-hydroxylase activity is critical in cortisol synthesis, whereas 17,20-lyase activity generates sex steroid precursors. The production of dehydroepiandrosterone (DHEA) by CYP17A1, from its precursor 17-hydroxypregnenolone (17-P5), is stimulated by cyt b_5. i. P450 cholesterol side-chain cleavage (P450scc); ii. 3β-hydroxysteroid dehydrogenase, Δ4,5-isomerase; iii. CYP17A1 (OHase); iv. CYP17A1 (lyase); v. 17β-hydroxysteroid dehydrogenase; vi. 5α-reductase; vii. aromatase (CYP19). (b) HPLC chromatograms of products formed upon incubation of human CYP17A1 with 17-P5 in the presence of human cyt b_5. Chromatographic mobility of DHEA, 17-P5 and pregnenolone (P5) standards are shown in top panel followed by a typical reaction in the absence of cyt b_5. Incubation of human CYP17A1 with increasing concentrations of ΔspMBP-cyt b_5-ApoAI* or in the presence of wild-type cyt b_5 are shown in the next set of panels. Chromatograms show substrate and product concentrations, measured as counts per minute (CPM), as a function of retention times.

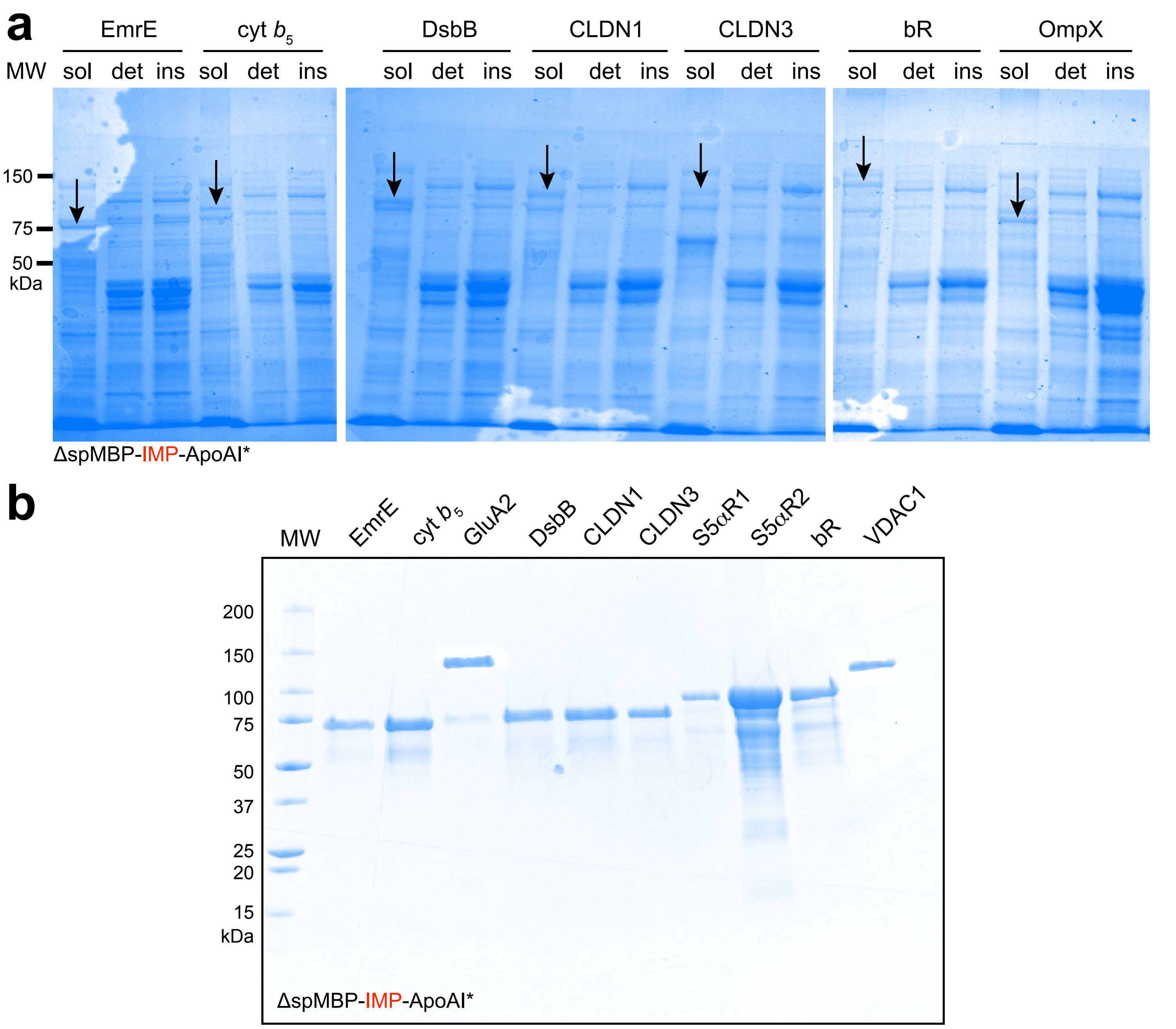

Supplementary Figure 6. *In vivo* solubilization of structurally diverse IMP targets. (a) Coomassie-stained gels of soluble (sol), detergent solubilized (det), and insoluble (ins) fractions derived from *E. coli* strain BL21(DE3) expressing representative ΔspMBP-IMP-ApoAI* fusions corresponding to the indicated IMPs. Molecular weight (MW) markers are shown on the left. (b) Coomassie-stained gel of purified fractions derived from *E. coli* strain BL21(DE3) expressing representative ΔspMBP-IMP-ApoAI* fusions corresponding to the indicated IMPs. Molecular weight (MW) ladder is shown on the left.

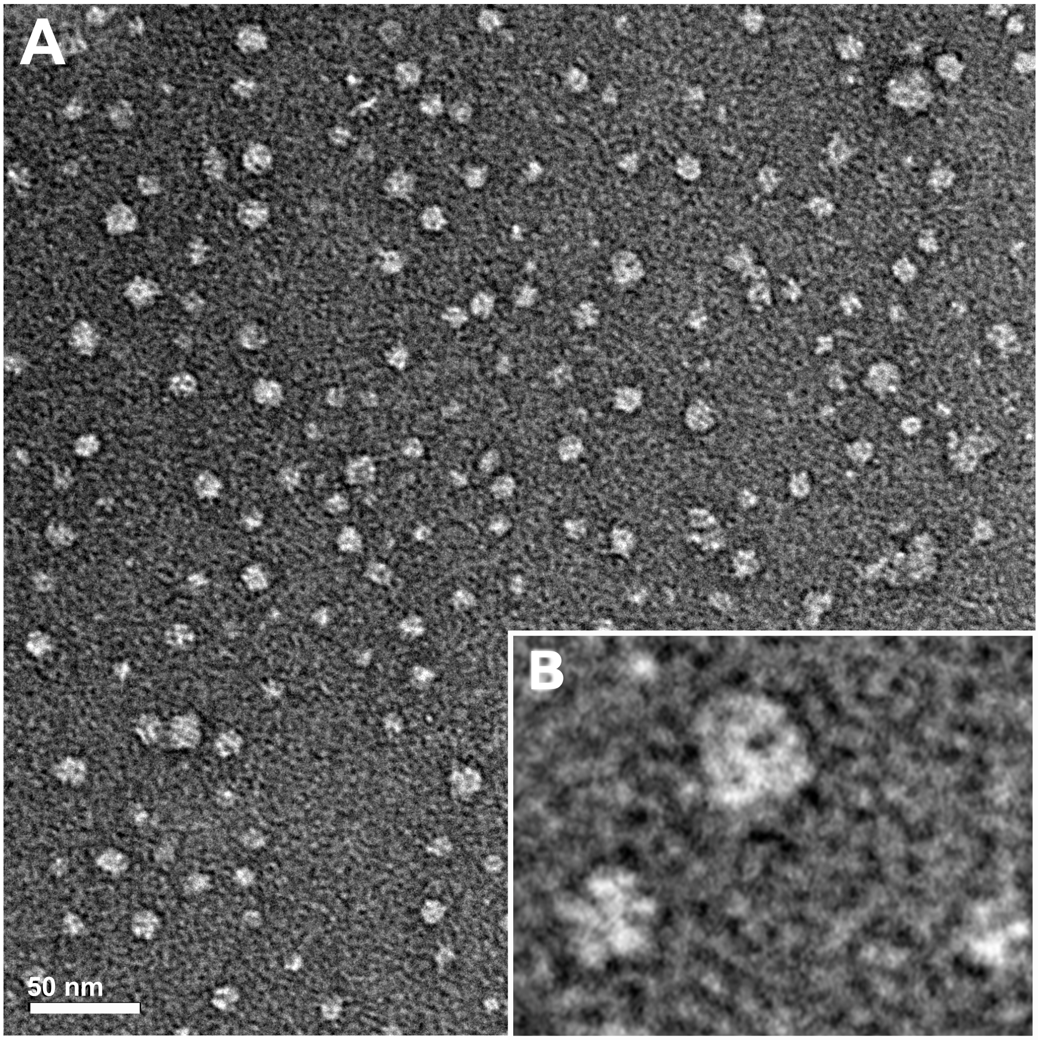

Supplementary Figure 7. Negative staining electron microscopy of solubilized EmrE. (A) Electron microscopy imaging by negative staining of purified OspA-EmrE-ApoAI* protein (0.1 mg/mL) prepared over 300-mesh carbon coated discs. The scale bar corresponds to 50 nm. (B) Amplification (5x) of OspA-EmrE-ApoAI* molecules from (A) generated using ImageJ software.

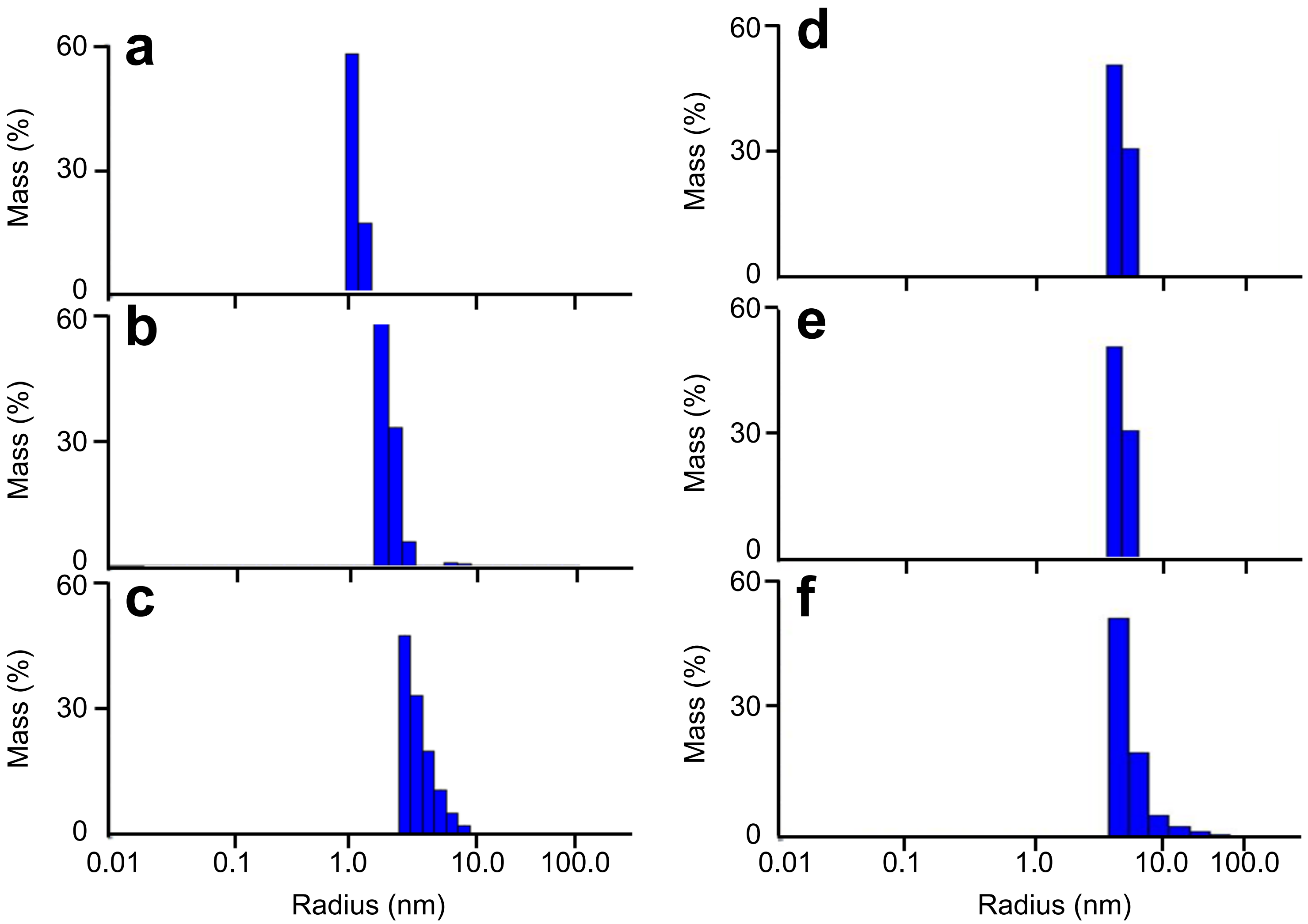

Supplementary Figure 8. Dynamic light scattering of SEC-purified OspA-EmrE-ApoAI*. (a-c) Dimeric OspA-EmrE-ApoAI* was incubated with 1 μM ethidium bromide (EtBr) and its size was monitored 5 min (b) and 15 min (c) after the mixing and compared to OspA-EmrE-ApoAI* in the absence of EtBr (a). (d-f) SEC-purified tetrameric OspA-EmrE-ApoAI* was incubated with 10 mM CHAPS (cmc 6-10 mM), a nondenaturing zwitterionic detergent for solubilizing membrane proteins and breaking protein-protein interactions. The distribution of protein size in solution was measured 15 min (e) and 60 min (f) after mixing and compared to tetrameric OspA-EmrE-ApoAI* in buffer without CHAPS (d). The *y*-axis represents the relative percentage of particles (mass %) of the total population of molecules in solution that exhibit a given radius (*x*-axis, in nm).

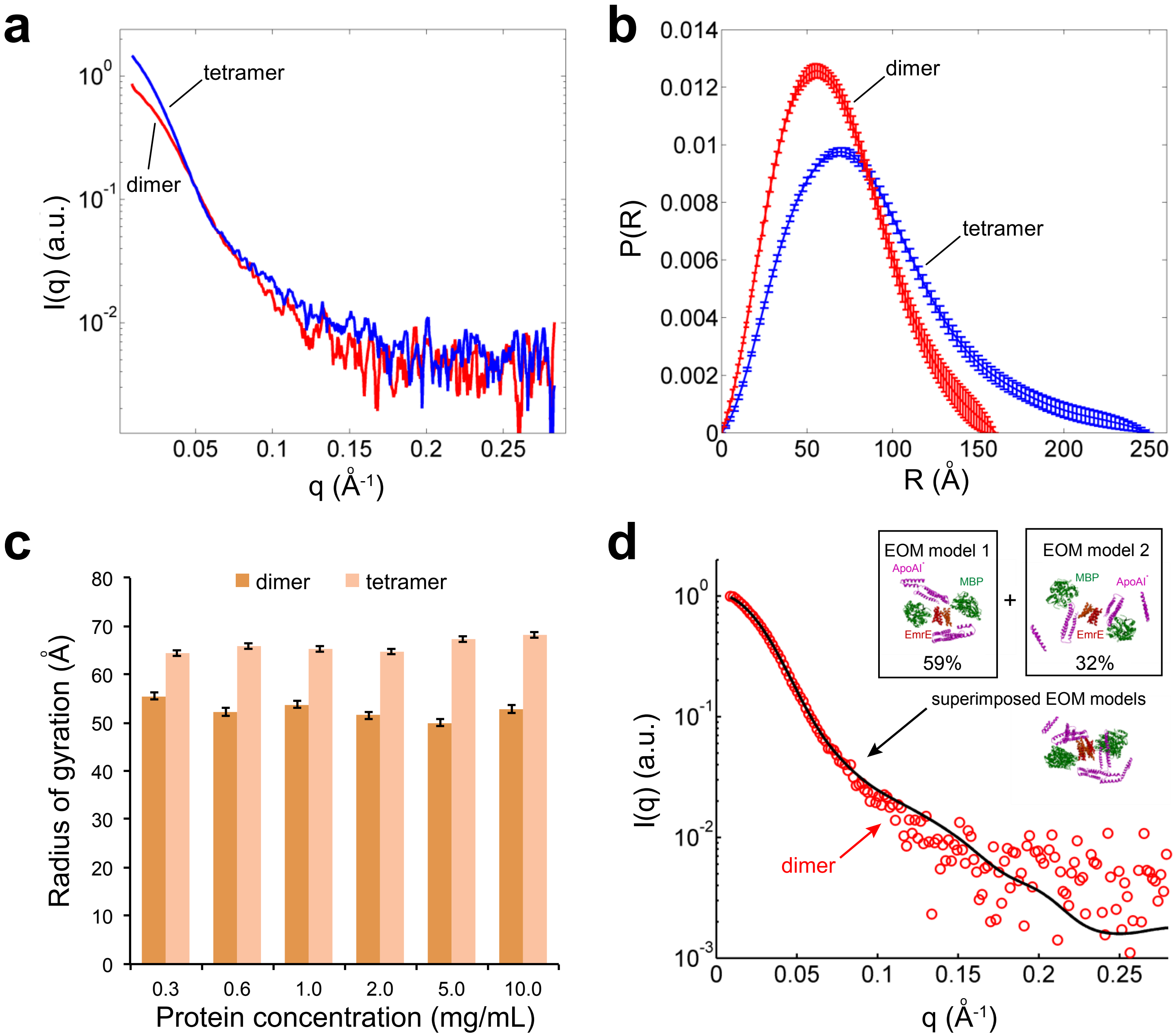

Supplementary Figure 9. Analysis of SAXS data for ΔspMBP-EmrE-ApoAI*. (a) Scattering intensity I(q) as a function of the scattering vector q measured at 1 mg/mL for both dimer and tetramer. The molecular mass is proportional to I(0), the extrapolated scattering intensity at zero scattering angle, hence the I(0) of ΔspMBP-EmrE-ApoAI* tetramer (1.6120 ± 0.0432) is nearly a factor of two of that of the dimer (0.8323 ± 0.0225). (b) The pair distance distribution function P(R) calculated by GNOM provides information about the particle shape and dimensions. The data demonstrates the differences in both the correlation length (peak position) and D_{max} (maximum dimension) between the dimeric (red) and tetrameric (blue) forms of ΔspMBP-EmrE-ApoAI*. (c) The radius of gyration for dimeric and tetrameric ΔspMBP-EmrE-ApoAI* species plotted as a function of protein concentration. The radius of gyration (the root mean square distance between the electrons within the particle) is the first-order structural parameter determined from the SAXS data, yielding direct information about the particle size. When a protein aggregates (*e.g.*, as a result of protein concentration), the radius of gyration increases. The results indicate that even at 10 mg/mL, ΔspMBP-EmrE-ApoAI* retains the radius of gyration observed at more dilute concentrations. (d) EOM fit to the scattering profile for ΔspMBP-EmrE-ApoAI* dimer. An ensemble containing three models was selected by EOM from a pool of 10,000 potential dimer structures to represent the experimental data (red circles). The two most populated models are shown inside the boxes along with their percentage populations. The averaged theoretical scattering profile of the ensemble (black line) was plotted against the dimer data, demonstrating good agreement ($\overline{\chi^2} = 0.250$). The superimposed models show that ApoAI proteins tend to wrap around the EmrE dimer while ΔspMBP lines up on the opposite sides of EmrE-ApoAI*.

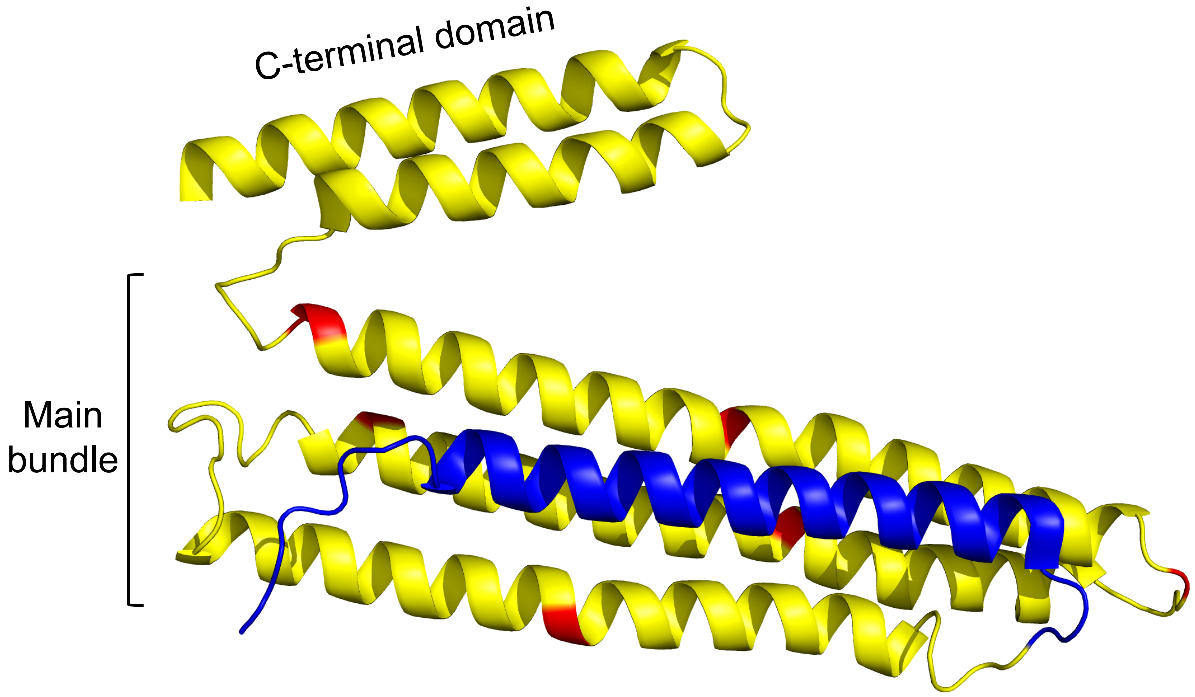

Supplementary Figure 10. Crystal structure of full-length ApoAI in lipid-free conformation. The ApoAI structure (Ajees et al., 2006 *Proc Natl Acad Sci USA*) is composed of two helical domains: the main bundle and the C-terminal domain. The first 43 amino acids (blue helix) are not part of the lipid-binding domain (yellow helices). In red are highlighted the residues (prolines and glycines) that in the absence of the N-terminal helix are responsible for breaking the helical structure and providing flexibility such that the structure can more easily interact with hydrophobic chains of lipids. This characteristic has been employed in the formation of nanodiscs.
